# Wings with stiffness and stroke variations enabling efficient flight and swimming for flapping-wing robots

**DOI:** 10.1093/nsr/nwag520

**Published:** 2026-08-22

**Authors:** Yang Xiang, Zeyu Gong, Chenyi Yang, Licheng Hou, Zhenwei Zhang, Yuhang Hong, Zhenfeng Gu, Le Gu, Ying Shi, Bo Tao, Han Ding

**Affiliations:** State Key Laboratory of Intelligent Manufacturing Equipment and Technology, Huazhong University of Science and Technology, Wuhan 430074, China; State Key Laboratory of Intelligent Manufacturing Equipment and Technology, Huazhong University of Science and Technology, Wuhan 430074, China; State Key Laboratory of Intelligent Manufacturing Equipment and Technology, Huazhong University of Science and Technology, Wuhan 430074, China; State Key Laboratory of Intelligent Manufacturing Equipment and Technology, Huazhong University of Science and Technology, Wuhan 430074, China; State Key Laboratory of Intelligent Manufacturing Equipment and Technology, Huazhong University of Science and Technology, Wuhan 430074, China; State Key Laboratory of Intelligent Manufacturing Equipment and Technology, Huazhong University of Science and Technology, Wuhan 430074, China; State Key Laboratory of Intelligent Manufacturing Equipment and Technology, Huazhong University of Science and Technology, Wuhan 430074, China; State Key Laboratory of Intelligent Manufacturing Equipment and Technology, Huazhong University of Science and Technology, Wuhan 430074, China; State Key Laboratory of Intelligent Manufacturing Equipment and Technology, Huazhong University of Science and Technology, Wuhan 430074, China; State Key Laboratory of Intelligent Manufacturing Equipment and Technology, Huazhong University of Science and Technology, Wuhan 430074, China; State Key Laboratory of Intelligent Manufacturing Equipment and Technology, Huazhong University of Science and Technology, Wuhan 430074, China

**Keywords:** bio-inspired, flapping-wing robot, aerial robot, aquatic robot, amphibious propulsion

## Abstract

Flapping-wing propulsion demonstrates high efficiency and maneuverability, commonly observed in natural species for either aerial flight or aquatic swimming. To chase prey and evade predators, biological flapping wings are specialized for a single medium, posing significant challenges in achieving effective and efficient amphibious flapping. Existing aerial-aquatic robots are primarily driven by artificial propulsion and lack bionic flapping-wing mechanisms. Meanwhile, current flapping-wing robots are operated only in a single medium, without amphibious propulsion capabilities. Inspired by the flapping principles of two different species, birds and manta rays, we design a bimodal flapping wing for both aerial and aquatic propulsion. The wing can switch between aerial and aquatic modes to adapt to the distinct requirements of air and water environments. In the aerial mode, metal tubular spars replicate the high stiffness of bird’s hollow bones, while limited rotation strokes of joints emulate asymmetric feather deformation. In the aquatic mode, elastic plates mimic the flexible cartilages of manta rays, and free rotation strokes of joints reproduce the symmetric fin deformation. Simplified analysis, dynamics tests, and robot motion tests have demonstrated that the wing’s aerial mode enhances lift generation during aerial flapping, while its aquatic mode decreases power consumption and improves thrust efficiency in aquatic flapping. Furthermore, by carrying auxiliary structures, the flapping-wing robot equipped with bimodal wings shows the potential to perform aerial-aquatic amphibious applications. Our work enhances the amphibious propulsion adaptability of large-scale flapping-wing robots and proposes a feasible engineering design.

## Teaser

Inspired by birds and manta rays, we develop a bimodal flapping wing that enables efficient aerial and aquatic propulsion, enhancing amphibious locomotion of large-scale flapping-wing robots.

## INTRODUCTION

Aerial-aquatic amphibious robots exhibit exceptional adaptability to diverse environments, possessing application prospects in biodiversity monitoring, environmental exploration, and mapping [1]. Although bionic flapping-wing propulsion demonstrates high efficiency and maneuverability in either aerial or aquatic environments [2,3], existing aerial-aquatic robots are typically driven by artificial propulsion, such as single propeller [4–6], multiple rotors [7–9], and jets [10,11]. An aerial-aquatic flapping-wing microrobot was proposed, capable of underwater motion and hovering using its wings, but its motion freedom and efficiency still leave room for improvement [12]. There are significant challenges to apply flapping-wing propulsion to aerial-aquatic environments [13], as the distinct medium parameters impose different requirements.

Currently, numerous aerial/aquatic flapping-wing robots have been developed for single-medium environments. Aerial flapping-wing robots typically exhibit stiff wing structures driven by brushless motors, enabling high flapping frequency to generate aerodynamics [14–16]. Additionally, an asymmetrical flapping stroke, which increases the wing’s projected area during downstroke and decreases it during upstroke, has been shown to enhance lift generation [17,18]. This approach has been validated to achieve greater payload and speed in aerial flapping-wing robots [19–21]. In contrast, aquatic flapping-wing robots utilize flexible wing structures driven by multiple servos. These robots exhibit lower flapping frequencies with more controllable degrees of wing surface, achieving a wave-like propulsion to decrease flapping resistance and improve efficiency [3,22]. Moreover, since buoyancy in the aquatic environment counteracts gravity, underwater flapping does not require additional lift. As a result, a nearly symmetrical flapping stroke is used to focus on generating thrust [23–25]. However, these aerial or aquatic flapping-wing robots show distinct design in driven mechanism, wing structures, and flapping strokes, making it difficult to directly integrate them for amphibious motions.

The real challenge in designing bimodal flapping wings lies in how to adaptively regulate the wing’s dynamics-generation mechanisms according to the distinct characteristics of air and water. Without specific amphibious design, existing aerial flapping wings will induce large flapping resistance underwater due to their large wing area and high stiffness. Moreover, the asymmetric stroke that enhances lift in air is not as essential for underwater propulsion. As a result, a large motor is required to drive aerial flapping wings underwater, which will increase the robot’s weight and size, thereby compromising flight stability. On the other hand, existing aquatic flapping wings struggle to meet lightweight requirements, and their flexible wing structures cannot capture enough airflow for flight. The symmetric stroke used in the water also causes lift loss in the air. Consequently, aquatic flapping wings alone are insufficient for stable flight.

The fundamental goal of biomimetic design is to understand how environmental constraints shape biological locomotion and to identify engineering trade-offs for robotic systems [26]. Since aerial flight requires continuous lift generation to counteract gravity, it imposes stringent requirements on structural lightweight and lift performance. Therefore, amphibious flapping wings should primarily prioritize maximizing lift and minimizing structural weight for flight, while also reducing underwater flapping resistance so that a lightweight motor can effectively drive the wings in amphibious environments. However, because flapping propulsion is generated by fluid reaction forces acting on the wings, reducing resistance may also weaken propulsion performance. Therefore, underwater swimming capability should be evaluated in terms of thrust efficiency (i.e. propulsion generated per power), to ensure that equivalent force generation is not significantly compromised after resistance-reduction design. Accordingly, an ideal bimodal flapping-wing design should maximize lift for flight while simultaneously reducing flapping resistance/improving thrust efficiency for swimming.

To achieve the bimodal wing design, we turn to nature for inspiration. Biological flapping wings are specialized to adapt to a single environment, evolving the ultimate performance for chasing prey and evading predators [27]. For instance, seabirds typically use their webbed feet to move underwater instead of their wings, while median and paired fin fish that efficiently swim underwater can only glide above the water for seconds. Although a few species, such as auks, have evolved the ability to fly and swim via their wings, this adaptation has resulted in them having the highest flight costs among seabirds [28]. Research indicates that the amphibious capabilities of auks arise from their distinct wing kinematics and geometries in different environments [29–31]. However, these differences are struggle for robotics to balance lightweight with complex function switching, that no auk-inspired robotic platform has yet been developed. It is challenging to achieve effective and efficient amphibious flapping inspired by a single species.

Thus, it is worth considering the integration of desirable phenotypic characteristics derived from different biological species. Birds and manta rays share a flapping form to move efficiently in aerial and aquatic environments, respectively [32,33]. Bird wings are composed of bones and feathers. Hollow bones exhibit a high stiffness in spanwise with lightweight, oscillating the wing rapidly to disturb airflow [34]. Feathers exhibit an asymmetric deformation stroke in chordwise to enhance lift [35], allowing air to flow through in upstroke while restricting air through during downstroke. For pectoral fins of manta rays, there are multiple radially oriented cartilages and cross-bracings to exhibit a flexibility in both spanwise and chordwise [36]. The flexible fins couple deformations with water and exhibit a symmetric deformation stroke during flapping [3], reducing flapping resistances and enhancing swimming efficiency. From functions of these biological tissues, the wing/fin differences between the two species can be categorized as: wing skeleton stiffness and deformation stroke. These studies inspire us that by switching stiffness and stroke, amphibious propulsion can be enhanced in flapping wings.

Due to the unique operating conditions of aerial-aquatic flapping, the design of variable stiffness/stroke structure faces multiple constraints. High-frequency aerial flapping generates strong vibrations, requiring the structures to possess sufficient strength and stability. Meanwhile, flapping flight is highly sensitive to wing weight. Excessive wing weight not only increases inertial impact during flapping, but also raises the lift required for sustained hovering. Therefore, strict lightweight design is essential. In addition, the wings are fully submerged during swimming which requires waterproof structural designs. Furthermore, bird bones/feathers differ substantially from the skeleton/cartilages of manta rays through natural evolution. Achieving both biomimetic morphology and functional compatibility under these design constraints remains highly challenging.

Based on the above considerations, this article presents an amphibious bimodal flapping wing with two motion modes, referred to as aerial and aquatic modes, mimicking the differing stiffness and strokes between bird wings and manta ray fins respectively (see Movie S1). By switching wing modes, dynamics tests demonstrate that the proposed bimodal flapping wings achieve a noticeable increase in lift generation during aerial flapping, as well as reduce resistance and improve thrust efficiency in aquatic flapping. The robot equipped with bimodal flapping wings achieves effective payload flight and efficient swimming. These results provide valuable insights for the future amphibious development of large-scale flapping-wing robots.

## RESULTS AND DISCUSSION

### Bio-inspired design principles

The overall configuration of amphibious flapping wings must balance the distinct propulsion mechanisms required in air and water. As discussed above, aerial flight imposes much stricter design constraints than underwater swimming. Therefore, amphibious flapping wings should primarily adopt a bird-inspired geometric layout to ensure sufficient dynamic interaction with airflow, while incorporating targeted bimodal designs to enhance lift in air and reduce resistance/improve efficiency underwater. A tubular spar is arranged along the spanwise direction as the primary driving skeleton, while multiple flexible rods are distributed chordwise to mimic feathers. By introducing a variable-stiffness joint into the spar and variable-stroke joints into the rods, the skeleton stiffness and deformation stroke of the wing can be adjusted, thereby regulating dynamics in amphibious environments.

Both variable-stiffness and variable-stroke joints fundamentally regulate the wing-deformation state during flapping, thereby influencing the propulsion forces generated by fluid–structure interactions. The deformation direction generally follows the direction of the fluid flow (see Fig. S1). For example, feathers undergo downward folding rotation under downward aerodynamic forces during upstroke. This compliant deformation characteristic allows the joints to be realized using lightweight and compact passive elastic actuators without additional servos or motors. Fluid forces drive the joints to undergo passive deformation, while the elastic potential energy stored in the joints enables rapid recovery during stroke reversal.

For the specific design of the variable-stroke and variable-stiffness joints, four key requirements must be considered: functional switching, lightweight, stability, and waterproof. Simpler mechanisms that more closely resemble biological morphologies and functions are better suited to satisfy these constraints simultaneously.

**Figure 1.**
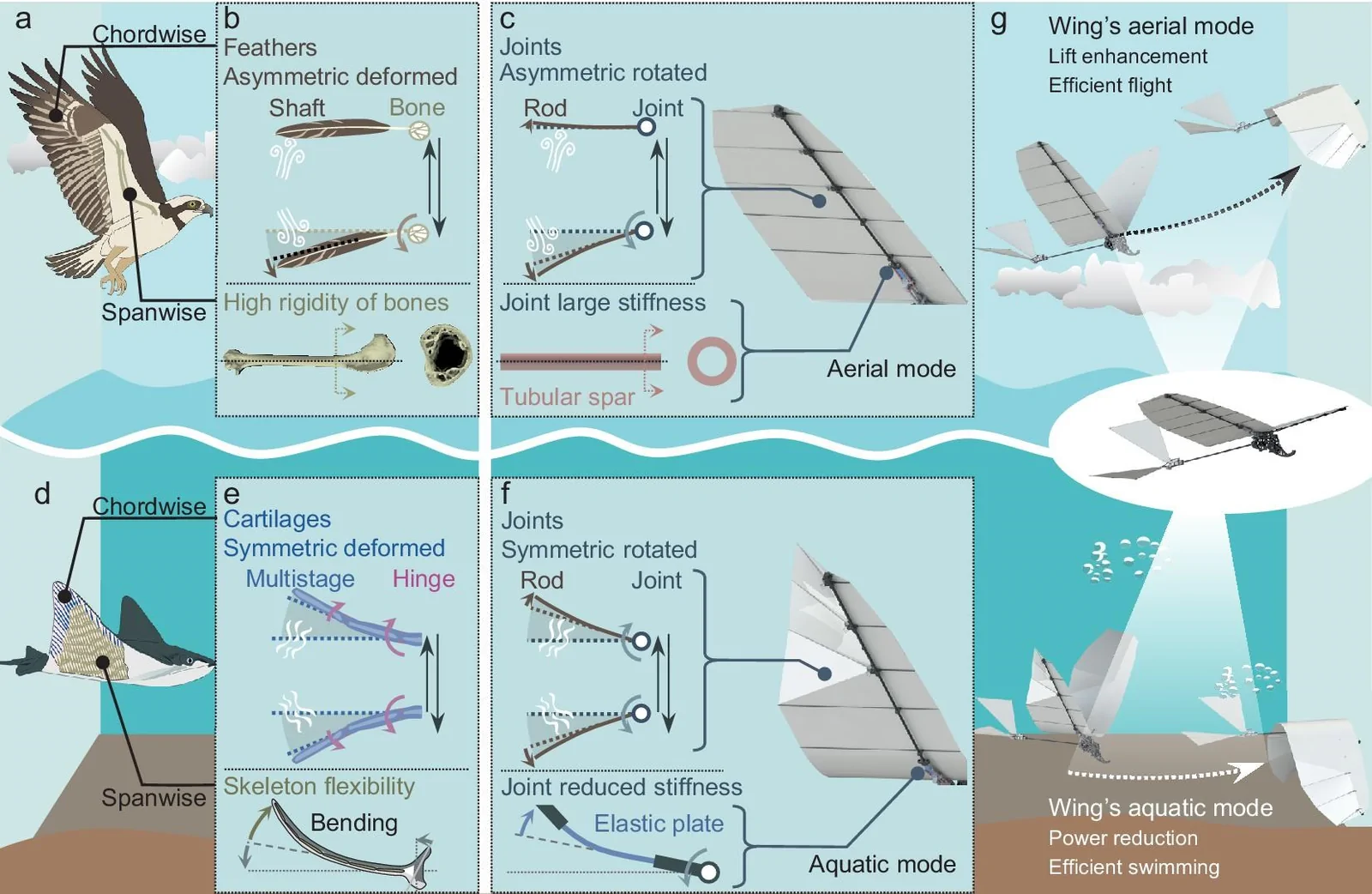
Biomimetic inspirations of the bimodal flapping wings. (a) Overall structural layout of a bird wing. (b) During flapping flight, the chordwise feathers undergo asymmetric deformation, while the spanwise bones remain highly rigid with negligible bending. (c) In the design, hinged joints with mechanical limits are used to reproduce asymmetric feather deformation, and hollow metal tubes are used to mimic the rigid bones. (d) Overall structural layout of a manta ray pectoral fin. (e) During flapping swimming, the chordwise cartilages exhibit approximately symmetric deformation, while the spanwise skeleton shows compliant bending. (f) In the design, hinged joints with released motion limits are used to reproduce symmetric deformation, and elastic plates are used to mimic compliant skeletal bending. (g) By switching wing modes, the robot enables lift enhancement in air and power reduction underwater for efficient amphibious propulsion.

The motion of bird feathers combines small-angle shaft deflection and large-angle root twisting, producing an asymmetric stroke with extension during downstroke and folding during upstroke (Fig. 1b). This mechanism can be simplified using flexible rods to mimic shaft deflection, hinged joints with mechanical limits to mimic asymmetric root twisting (Fig. 1c), and torsional springs to provide passive elastic response. Likewise, the hollow, lightweight, yet highly rigid bird bones can be mimicked using high-stiffness metal tubes.

The pectoral fins of manta rays consist of hinged cartilage arrays. During flapping, the inner cartilages exhibit approximately symmetric large-angle twisting, while the twisting amplitude decreases toward the fin edge (Fig. 1e). This mechanism can similarly be simplified using flexible rods and hinged joints with released motion limits to achieve symmetric stroke (Fig. 1f). In addition, the compliant bending of the manta ray skeleton can be mimicked using elastic plates, enabling passive deformation and recovery through stored elastic energy. All joint materials are selected to support direct underwater operation for waterproof performance. These principles provide effective biomimetic correspondence in both morphology and function.

However, once the elastic parameters of the joints are selected, they cannot be adjusted in real time during operation. Therefore, the specific elastic values must be optimized according to the flight and swimming conditions. Torsional springs can be used for elastic rotation in chordwise variable-stroke joints, while the rigid tubes and elastic plates can be used for compliant bending in spanwise variable-stiffness joints. The dynamics under different elastic configurations should be systematically analyzed and experimentally evaluated to ensure optimal amphibious propulsion of the bimodal wings.

### Design of the bimodal flapping wing

**Figure 2.**
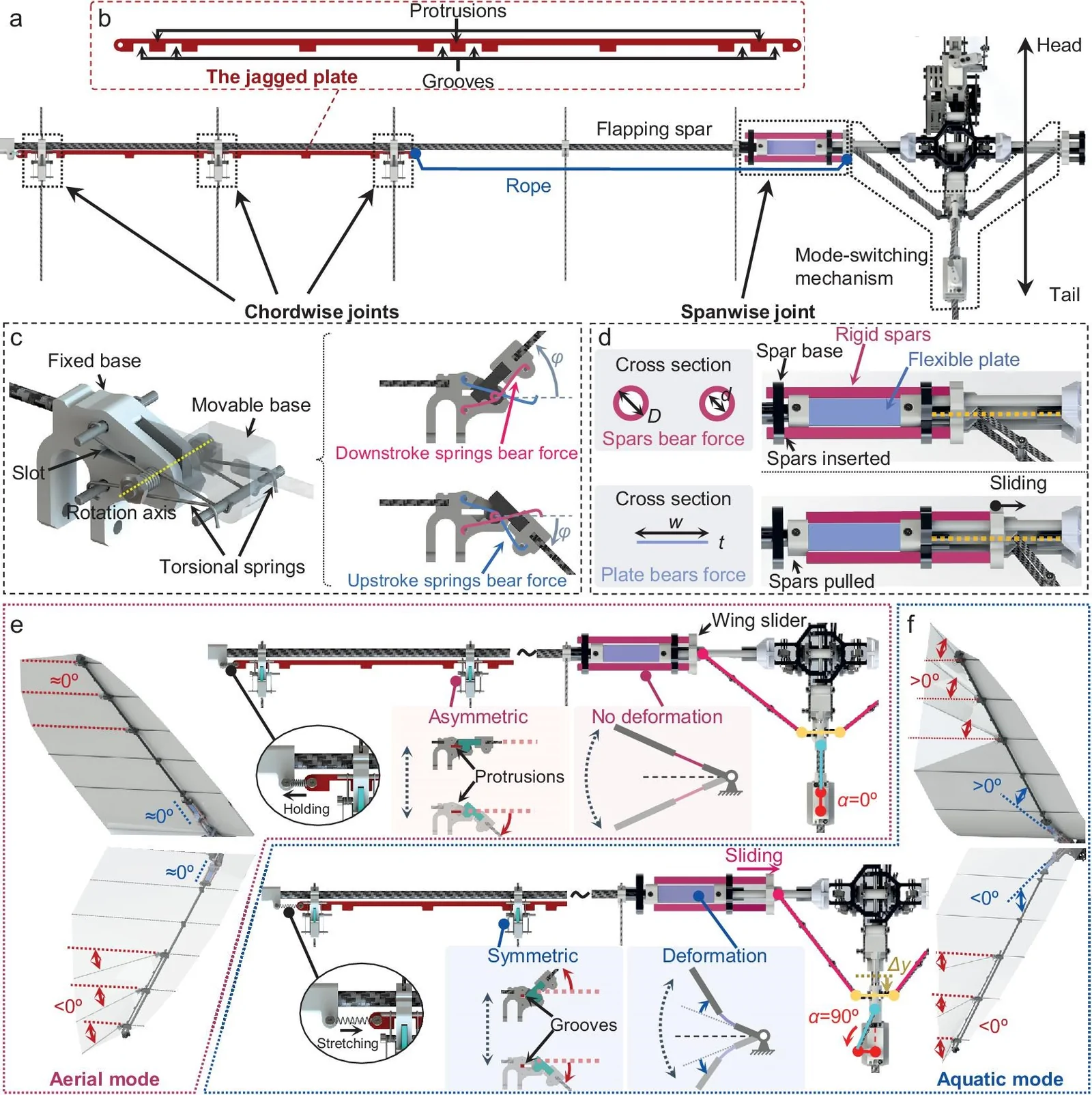
Structures and mode-switching principles of the bimodal flapping wing. (a) Structural layout of the bimodal flapping wing with the mode-switching mechanism. (b) The jagged plate exhibits multiple protrusions and grooves. (c) The movable base of the chordwise joint can rotate relative to the fixed base with a range of [*-45*°, *45*°], while torsional springs manage dynamical response of the chordwise joint. (d) A flexible plate connects the spar base and wing root of the spanwise joint, two rigid spars are driven by the wing slider to be inserted into or pulled from the spar base. (e) Demonstration of wing aerial mode: rigid spars bear the wing load in the spanwise joint, while protrusions of the jagged plate are in the chordwise joints, resulting in stiffer bending stiffness with asymmetric strokes (see Movie S2). (f) Demonstration of wing aquatic mode: flexible plate bears the wing load in the spanwise joint, while grooves of jagged plate are in the chordwise joints, producing softer bending stiffness with symmetric strokes (see Movie S2).

Based on the above biomimetic principles, this section presents the implementation of the bimodal flapping wings. As shown in Fig. 2a, a flapping spar actively drives the wing motion, while five chordwise rods undergo passive deformation during flapping. Since the outer wing regions contribute most significantly to propulsion generation in both birds and manta rays [3,37], variable-stroke joints are only integrated into the three rods near the wingtip to reduce structural complexity and weight, while the two root-side rods are fixed directly to the spar (see Figs S2 and S3). A variable-stiffness joint near the wing root transmits bending deformation across the wing surface, while a fuselage-mounted mode-switching mechanism synchronously switches the modes of both wings.

A jagged plate with multiply protrusions and grooves is positioned near the wingtip (Fig. 2b), passing through the three chordwise joints. One end of the plate is connected to the wingtip via a small spring, and the other end is connected to the mode-switching mechanism via a rope. Pulling or releasing the rope can determine whether the protrusions or grooves are in chordwise joints, thereby altering the joint between asymmetric and symmetric strokes.

The chordwise variable-stroke joint with a weight of 5 g is detailed in Fig. 2c, consisting of a fixed base, a movable base, and four torsional springs. The fixed base is set on the flapping spar, and a slot is designed in the base to accommodate the passage of the jagged plate. While the movable base allows a passive rotation angle *φ* relative to the fixed base within [*-45*°, *45*°], this range can be adjusted by the jagged plate to realize asymmetric or symmetric rotation. The joint rotation mimics feather twisting and fin deformation, while the attached rod further reproduces the small deflection of feather shafts and edge cartilages. Four torsional springs provide passive dynamic response during flapping, coupling the rotation angle *φ* with fluid loading. Different chordwise stiffness $\kappa _c$ can be achieved by changing the spring parameters. The joint mainly consists of waterproof 3D-prints, carbon plates, and metal torsional springs, enabling direct underwater operation.

The spanwise variable-stiffness joint with a weight of 40 g is shown in Fig. 2d, consisting of a flexible plate, two rigid spars, a spar base, and a wing slider. The flexible plate mimics the compliant bending of manta ray skeleton, while the rigid spars replicate the high stiffness of bird bones. Driven by the mode-switching mechanism, the wing slider moves toward either the wing tip or root. When the slider moves toward the wingtip, the rigid spars are inserted into the spar base, increasing the bending stiffness of the wing skeleton. When the slider moves toward the wing root side, the rigid spars are withdrawn, leaving the flexible plate to provide compliant bending with lower stiffness. The joint mainly consists of waterproof 3D-prints, carbon plates, and metal components, enabling direct underwater operation.

### Switching between the aerial and aquatic modes

Since the left and right wings must switch modes synchronously during amphibious operation, a lightweight linkage mechanism driven by a single servo is adopted as the mode-switching mechanism (see Fig. S4). This design avoids the additional weight of multiple actuators while ensuring stable and reliable force transmission. The mechanism converts the servo rotation into the sliding motion of the wing sliders without interfering with wing flapping. The rigid spars of the spanwise joints and the ropes of the chordwise joints are both connected to the wing sliders and move synchronously during mode switching. The specific mode-switching principle is described as follows (see Movie  S3).

*Aerial mode*. When the mode-switching mechanism is in the initial position (Fig. 2e), the wing slider is positioned near the wingtip side. At this point, the rigid spars are inserted in the spar base at the spanwise joint, resulting in a stiffer skeleton stiffness like bird bones with negligible spanwise deformation during flapping. Meanwhile, the rope connected to the jagged plate is released, causing the jagged plate to be held in position by the spring at the wingtip. Protrusions of the jagged plate engage the chordwise joints, limiting the rotation angle of the movable base to an asymmetric stroke range of [*-45*°, *0*°].

*Aquatic mode*. When the servo of mode-switching mechanism rotates to *90*° (Fig. 2f), the wing slider moves toward the wing root. This motion pulls the rigid spars out of the spar base in spanwise joint, allowing the flexible plate to bear the entire wing load. As a result, the spanwise joint exhibits a softer bending stiffness akin to the flexible skeleton of manta rays, enabling the wing to bend during flapping. At the same time, the rope pulls the jagged plate toward the wing root, causing the grooves of the jagged plate to fit into chordwise joints. The movable based of chordwise joints are thereby released to perform a symmetric stroke within the range of [*-45*°, *45*°]. The spring connecting the jagged plate and the wingtip is stretched, generating a restoring force that tends to return the jagged plate to its initial position.

When the servo rotates back to *0*°, the rigid spars are reinserted into the spar bases, while the stretched wingtip springs release stored elastic energy to return the jagged plates to their initial positions. In this way, the wings can repeatedly switch between aerial and aquatic modes. Since only small wingtip springs are required to maintain the position of the jagged plate, the power consumption for mode switching is negligible and has little impact on the robot’s overall endurance (see Fig. S5).

### Simplified analysis of different joint stroke and stiffness

Since flapping motion involves complex three-dimensional fluid–structure interaction, it is challenging to achieve a perfect theoretical analysis of flapping dynamics. However, simplified models can be employed to investigate the relative influence trends of structural and kinematic parameters on dynamic performance and to identify approximate optimal parameter ranges. Here, we analyze the dynamic performance of chordwise variable-stroke and spanwise variable-stiffness joints based on the torque balance principle during the flapping process.

To simplify the analysis of how chordwise joint strokes affect flapping dynamics, two assumptions are made based on existing flapping dynamics calculation methods: (i) the wing is assumed to be rigid, with only the deformation induced by the chordwise joint considered [38]; (ii) the cross-section at 3/4 of the wing semi-span is selected as the equivalent plane to represent the overall semi-wing deformation [20].

The theoretical coordinate system and schematic used for chordwise joints are shown in Fig. S7 of the supplementary information. The *x*-axis is defined along the rotatable wing chord, where *x* = *-1* corresponds to the rotation point of chordwise joint and *x* = 1 corresponds to the trailing edge. The kinematic description *h(x,t)* of the equivalent cross-section during flapping is given as


(1) \begin{eqnarray*}
h(x,t) = \frac{3D}{2c}{\rm sin}\, \theta +(x+1){\rm sin}\, \phi ,
\end{eqnarray*}

where *c* is the chord length of the wing that can rotate with the joint, *D* is the semi-span length, *θ (t)* is the flapping angle, *t* is time, and *ϕ (t)* is the passive chord rotation angle. We simulated the chordwise joint motion during flapping by setting two parameters: (i) The initial angle of chordwise joint $\phi _0$, determines the offset of the joint motion stroke. (ii) The torsional spring stiffness of chordwise joint $\kappa _c$, affects the dynamic response of the joint. According to the reference [38], the passive chordwise stroke follows the torque balance principle:


(2) \begin{eqnarray*}
J_c \ddot{\phi }= -\kappa _c(\phi -\phi _0) + N_{fc} + N_{ic},
\end{eqnarray*}

where $J_c$ denotes the rotational inertia of the chord, $N_{fc}$ represents the moment exerted on the chordwise section by the fluid, and $N_{ic}$ denotes the moment due to flapping acceleration on the chord section. We define a fluid domain as a unit circle centered at *x* = 0, while an acceleration potential $\mathcal {G} = \varphi + i\psi$ exists in this domain [39]. *φ* is Prandtl’s acceleration potential, as grad *φ* gets the acceleration field of the flow. *ψ* is defined by the Cauchy–Riemann equations. Lift, thrust, and power are calculated under different parameters by solving the potential. Given the overall length of the main text, detailed derivations and solution processes are derived in the supplementary information.

Similarly, the influence of the spanwise joint bending stiffness on dynamic performance can be analyzed using the same simplified calculation framework introduced above. Two assumptions are applied in the calculation: (i) the wing is assumed to be rigid, with only the deformation induced by the spanwise joint considered; (ii) the cross-section at the flapping spar is selected as the equivalent plane to represent the overall semi-wing deformation.

The theoretical coordinate system and schematic used for spanwise joints are shown in Fig. S8 of the supplementary information. The $x_o$-axis is defined along the outer segment span, with $x_o$ = *-1* at the junction between the outer segment and the spanwise joint, and $x_o$ = 1 at the tip of outer segment. During flapping, the bending of the spanwise joint is represented by a cantilever-beam model. The beam coordinate $\lbrace x_b, \delta \rbrace$ is defined as $x_b$ along the inner segment span and *δ* normal to the inner-span direction. The kinematic equation $h(x_o, t)$ of the outer segment during flapping can be expressed as


(3) \begin{eqnarray*}
h(x_o, t) &=& (x_o + 1){\rm sin}(\tau _e + \theta ) \\
&& +\, \frac{2(D_1+D_2)}{D_3}{\rm sin}\theta + \tilde{\delta }_e {\rm cos}\,\theta ,
\end{eqnarray*}

where the driving spar of the wing is divided into three segments: $D_1$ is the inner segment length, $D_2$ is the variable-stiffness joint length, and $D_3$ is the outer segment length. $\tilde{\delta }_e$ represents the nondimensional tip deflection of the spanwise joint under cantilever-beam model, and $\tau _e$ is the tip bending angle of the joint. During flapping, the spanwise joint should also satisfy the moment balance among the elastic torque generated by bending, the inertial torque from flapping, and the fluid-induced torque. The balance equation of spanwise joint can be formulated as


(4) \begin{eqnarray*}
J_s \ddot{\tau }_e = -N_e + N_{fs} + N_{is},
\end{eqnarray*}

where $J_s$ is the rotational inertia of the outer segment, $N_e$ is the equivalent bending moment of external loading on the spanwise joint, $N_{fs}$ represents the moment exerted on the spanwise section by the fluid, and $N_{is}$ denotes the moment due to flapping acceleration on the section. We also define a fluid domain as a unit circle centered at $x_o$ = 0 with an acceleration potential $\mathcal {G} = \varphi + i\psi$. The detailed process of solving the potential and dynamics are shown in the supplementary information.

Based on the above simplified theoretical analysis, the dynamics of the chordwise and spanwise joints are summarized at an aerial flapping frequency of *f* = 3 Hz and an aquatic flapping frequency of *f* = 0.15 Hz (see Figs S7 and S8). The results indicate that, for the chordwise joint, the asymmetric stroke with $\kappa _c$ = 0.030 N m achieves the largest lift in air, while the symmetric stroke with $\kappa _c$ = 0.440 N m provides the best thrust underwater. For the spanwise joint, the dynamic performance no longer increases significantly when the bending stiffness $\kappa _s \ge$ 9 N m$^2$ in air, whereas underwater flapping achieves the highest thrust efficiency at $\kappa _s$ = 5 N m$^2$.

It should be noted that the proposed simplified theoretical analysis partially differs to some extent from the actual motion state, and its quantitative accuracy is subject to some limitations. Nevertheless, the model remains effective for evaluating the relative influence of different joint parameters. These results validate the role of variable stiffness and stroke in modulating flapping dynamics, and provide preliminary guidance for subsequent experiments to determine optimal joint elastic parameters.

### Dynamics tests of different joint stroke and stiffness

To experimentally compare dynamics of different wing modes and analyze optimal elastic parameters of joints, we organize a series of dynamics tests in both air and water (see Fig. S10 and Movie  S4). These tests measure forces, power, and efficiencies under various stroke and stiffness configurations. Lift *L* and thrust *T* are measured by a 6-axis force/torque sensor during testing, while power consumption *P* is recorded via a current sensor. Lift efficiency $\eta _L$ and thrust efficiency $\eta _T$ are defined as the force induced per watt of power consumed. The flapping angle *θ* is set to a range of [−15°, 40°] in both air and water. Noticeably, a pair of wings is tested in the air, while only a side of wings is submerged into water for underwater tests.

The structural variables of chordwise joints are the rotation stroke and torsional spring stiffness $\kappa _c$, while those of spanwise joints are the bending stiffness $\kappa _s$ of rigid spars and flexible plates. Based on previous design principles, the evaluation criteria for test results are established as: (i) in the air, the goal is achieving large lift; (ii) in the water, the focus is on reducing power consumption while enhancing thrust efficiency.

**Figure 3.**
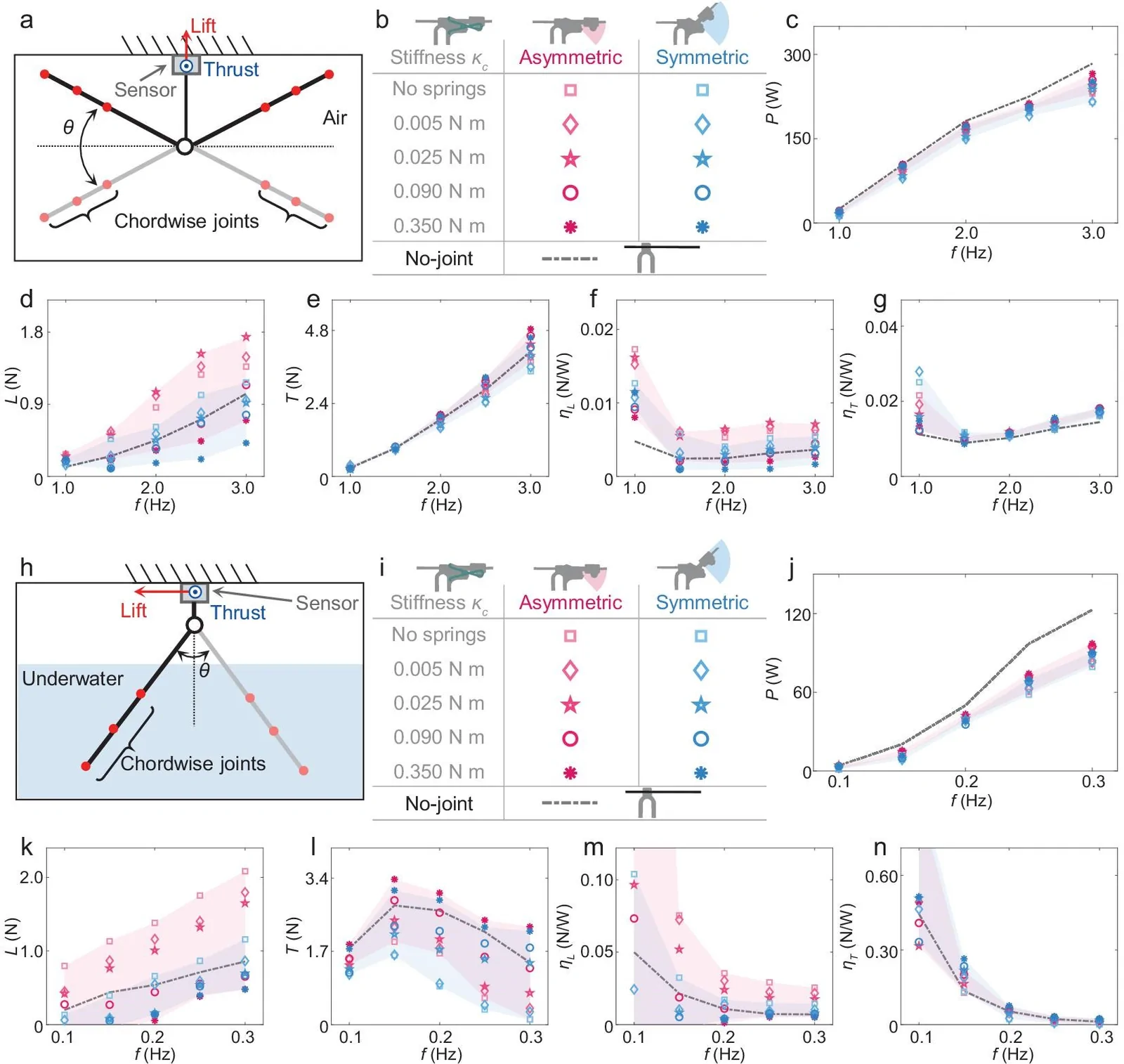
Dynamics of chordwise joints under different rotation stroke and spring stiffness. (a) A pair of wings with chordwise joints is fixed to a 6-axis force/torque sensor in air, measuring data over a flapping-frequency range of 1.0–3.0 Hz. (b) Two strokes were tested with torsional spring stiffness ranging from no-spring to 0.350 N m, as well as under a no-joint condition (the black dashed lines). (c) Measured power consumption during aerial flapping. (d) Measured lift data during aerial flapping. (e) Measured thrust data during aerial flapping. (f) Measured lift efficiency during aerial flapping. (g) Measured thrust efficiency during aerial flapping. (h) The semi-wing with chordwise joints is fixed to a pool through the 6-axis force/torque sensor, submerging into the water and measuring data over a flapping-frequency range of 0.1–0.3 Hz. (i) Two strokes were tested with torsional spring stiffness ranging from no-spring to 0.350 N m, as well as under a no-joint condition (the black dashed lines). (j) Measured power consumption during aquatic flapping. (k) Measured lift data during aquatic flapping. (l) Measured thrust data during aquatic flapping. (m) Measured lift efficiency during aquatic flapping. (n) Measured thrust efficiency during aquatic flapping. See Fig. S13 for the statistical analysis of the results.

As depicted in Fig. 3, no-springs and four torsional springs ($\kappa _c$ from 0.005 to 0.350 N m) combined with two strokes (symmetric and asymmetric) are tested for chordwise joint analysis. To specifically investigate the dynamic adjustments induced by chordwise joints, the spanwise joints are fixed with a bending stiffness of 20.5 N m$^{2}$. Additionally, a no-joint mode, which can be regarded as the chord directly fixed to the flapping spar, is also tested to demonstrate the advantages of joints. Results indicate that asymmetric stroke exhibits a significant advantage in lift generation during aerial flapping, while symmetric stroke reduces power consumption and enhances thrust efficiency during aquatic flapping. Using maximum lift in air and maximum thrust efficiency in water as the optimization criteria, the optimal torsional spring stiffness is determined to be 0.025 N m with asymmetric stroke for aerial flapping and 0.350 N m with symmetric stroke for aquatic flapping.

However, in practical structural design, it is challenging to switch torsional spring stiffness of chordwise joints under structural stability and lightweight requirements. Meanwhile, considering that aerial flight demands a higher flapping frequency and stricter dynamic performance than aquatic swimming, the design must prioritize aerial flight requirements. Therefore, we choose the spring stiffness of chordwise joints as 0.025 N m for both aerial and aquatic environments, scarifying around *33%* thrust and thrust efficiency in aquatic environment but streamlining structural design.

**Figure 4.**
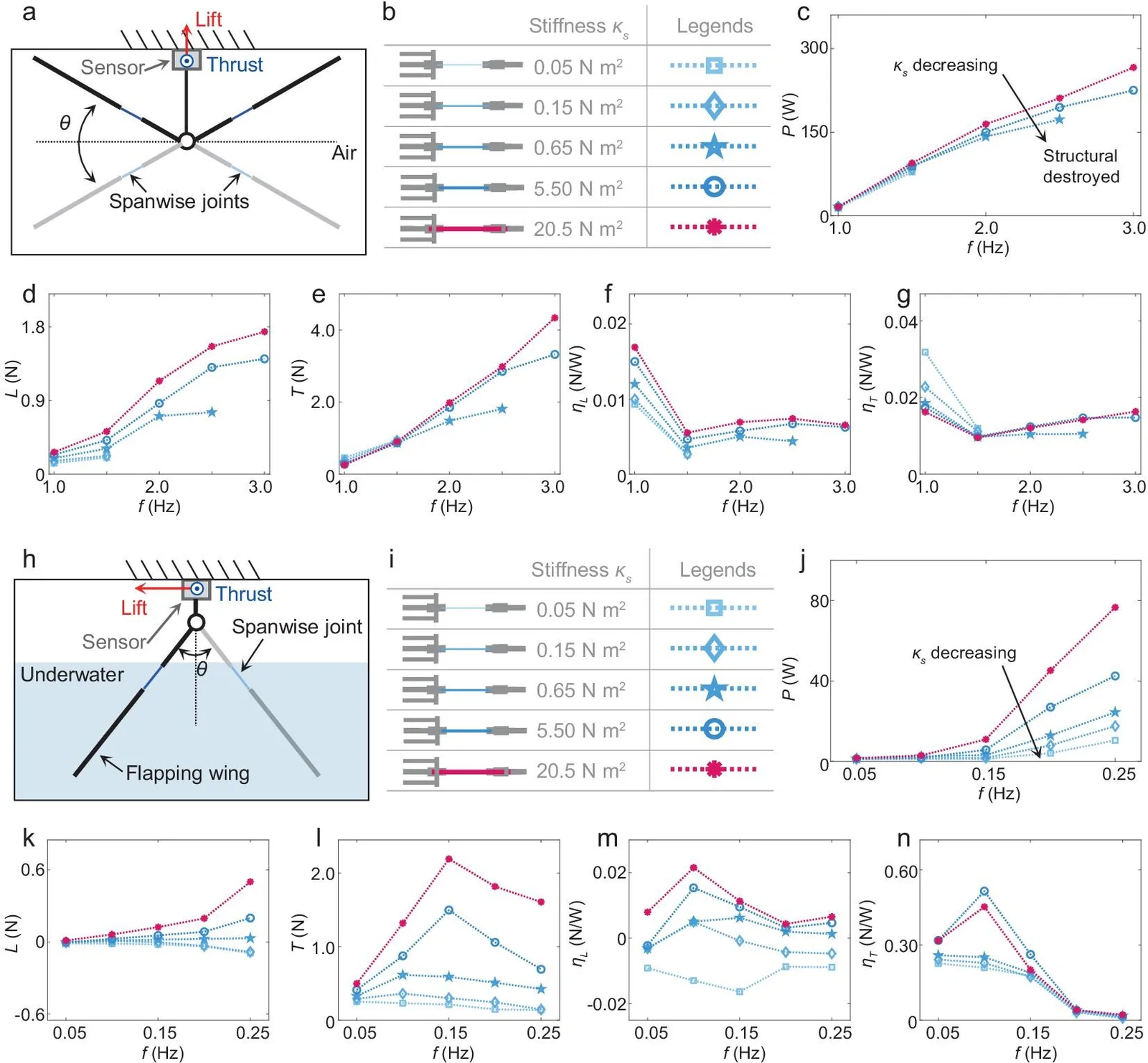
Dynamics of spanwise joints under different bending stiffness. (a) A pair of wings with chordwise joints is fixed to a 6-axis force/torque sensor in the air, measuring data over a flapping-frequency range of 1.0–3.0 Hz. (b) Five bending-stiffness values are tested within a range of *[0.05, 20.5]* N m$^{2}$. (c) Measured power consumption during aerial flapping. (d) Measured lift data during aerial flapping. (e) Measured thrust data during aerial flapping. (f) Measured lift efficiency during aerial flapping. (g) Measured thrust efficiency during aerial flapping. (h) The semi-wing with spanwise joint is fixed to a pool through the 6-axis force/torque sensor, submerging into the water and measuring data over a flapping-frequency range of 0.05–0.25 Hz. (i) Five bending stiffness are tested within a range of *[0.05, 20.5]* N m$^{2}$. (j) Measured power consumption during aquatic flapping. (k) Measured lift data during aquatic flapping. (l) Measured thrust data during aquatic flapping. (m) Measured lift efficiency during aquatic flapping. (n) Measured thrust efficiency during aquatic flapping. See Fig. S13 for the statistical analysis of the results.

In subsequent dynamics tests for spanwise joints (Fig. 4), we set the spring stiffness of chordwise joints to 0.025 N m, asymmetric chordwise stroke is employed in aerial tests while symmetric chordwise stroke is used in aquatic tests. Five spanwise bending stiffness $\kappa _s$, ranging from 0.05 to 20.5 N m$^{2}$, are tested in both air and water. During aerial flapping, results indicate that a spanwise bending stiffness of 20.5 N m$^{2}$ captures the largest forces but consumes most power, while an excessive spanwise flexibility (<0.65 N m$^{2}$) causes structural failure when the flapping frequency exceeds 2.0 Hz. In aquatic flapping, 20.5 N m$^{2}$ bending stiffness achieves the largest forces and lift efficiency, while a stiffness of 5.50 N m$^{2}$ exhibits the highest thrust efficiency and significant reduces power consumption.

These results indicate that a higher spanwise bending stiffness favors aerodynamics generation during flight, whereas an appropriate degree of spanwise flexibility reduces power consumption and enhances thrust efficiency during swimming. Taking maximum aerial lift and maximum aquatic thrust efficiency as the optimization criteria, the optimal bending stiffness of the spanwise joint is determined to be 20.5 N m$^{2}$ for aerial flapping and 5.50 N m$^{2}$ for aquatic flapping. Representative time-varying data of the dynamic tests are provided in Figs S11 and S12.

It should be noted that dynamic tests were conducted in still air and water. Although this method is widely used to evaluate the relative effects of structural parameters on flapping performance [16,25], it differs from the oncoming flow conditions during actual flight and swimming. The oncoming flow generates additional aerodynamic/hydrodynamic forces on the wing based on the Bernoulli principle and Newton's third law, producing a restoring moment that tends to return the deformed joints toward their initial positions. As a result, the optimal elastic parameters in real operating conditions may be slightly lower than those identified in still-flow tests. However, given the relatively low operating velocities of the robot (*≤*6 m s$^{-1}$ in air and *≤*0.3 m s$^{-1}$ in water), the influence of oncoming flow on wing deformation is expected to be limited. Therefore, the optimal parameters obtained from still-flow tests remain a reasonable reference for practical applications.

The experimental results are generally consistent with theoretical predictions (see Fig. S9). Both indicate that an asymmetric chordwise stroke combined with high spanwise bending stiffness enhances positive lift generation in air, whereas a symmetric stroke with appropriate spanwise flexibility reduces underwater flapping resistance and improves thrust efficiency. Experimentally, the optimal elastic parameters are $\kappa _c$ = 0.025 N m with $\kappa _s\ge$ 12 N m$^2$ for aerial flapping, and $\kappa _c$ = 0.350 N m with $\kappa _s$ = 5.50 N m$^2$ for aquatic flapping. Theoretically, they are $\kappa _c$ = 0.030 N m with $\kappa _s\ge$ 9 N m$^2$ for aerial flapping, and $\kappa _c$ = 0.440 N m with $\kappa _s$ = 5.50 N m$^2$ for aquatic flapping. These results cross-validate the effectiveness of the proposed variable-stroke and variable-stiffness joints and the reliability of the selected optimal parameters.

Compared to traditional flapping wings (no-joint mode) in aerial flapping, at a flapping frequency of 2.5 Hz, the aerial mode of the bimodal flapping wings not only achieves a decrease of *8%* in power consumption, but also achieves a *119%* increase in lift, *134%* in lift efficiency, and *11%* in thrust efficiency. Simultaneously, in aquatic flapping with a flapping frequency of 0.15 Hz, the aquatic mode exhibits a *72%* reduction in power consumption and a *46%* decrease in thrust compared to the no-joint mode, resulting in a *94%* improvement in thrust efficiency. These findings highlight that, although the bimodal flapping wings introduce additional structural complexity, the design tailored to the distinct dynamic requirements of aerial flight and aquatic swimming enables superior dynamic performance compared to traditional flapping wings.

### Propulsion performance of the bimodal wing

We further mounted the bimodal flapping wings onto the robotic platform and conducted motion tests to evaluate the effects of different modes on propulsion performance (see Movie S5). To evaluate the effect of wing mode switching on propulsion, the robot equipped with bimodal flapping wings is set to move along a straight path, with its position and forward velocity measured using a motion capture system (see Fig. S14). Moreover, the robot’s flight payload capacity and swimming power consumption are utilized to demonstrate the lift and efficiency advantages of the bimodal wing design.

**Figure 5.**
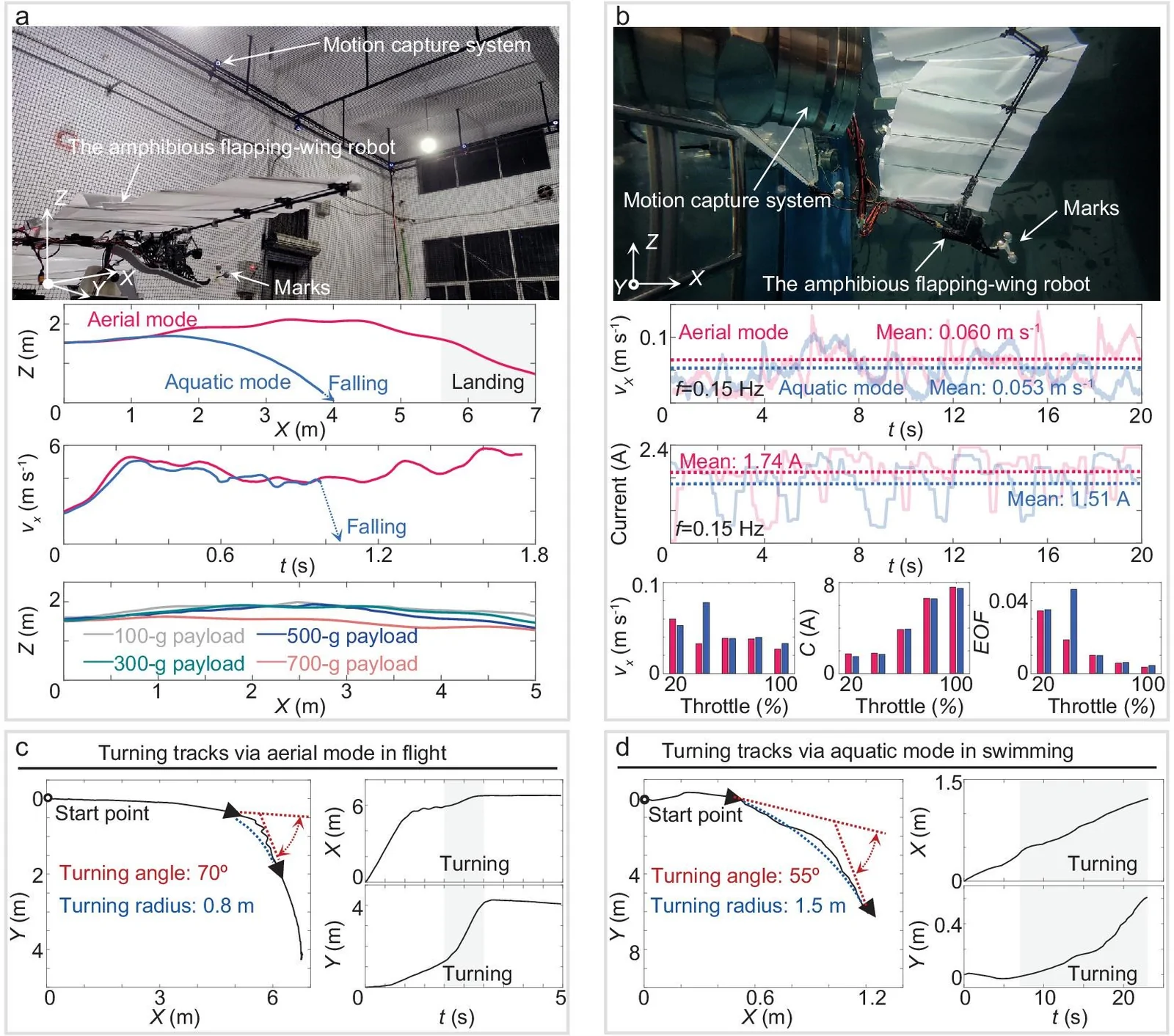
Propulsion performance of the aerial and aquatic modes during amphibious free motions. (a) The wing’s aerial mode enables the 850-g robot to achieve stable forward flight with a 700-g payload. (b) The wing’s aquatic mode exhibits lower power consumption while maintaining propulsion efficiency during swimming. (c) In the aerial mode, the wing’s propulsion allows the robot to execute effective tail-assisted turns during flight. (d) In the aquatic mode, the wing’s propulsion similarly enables effective tail-assisted turns during swimming.

In the aerial flight tests, at a flapping frequency of 3.00 Hz, the wing’s aerial mode enables the 850-g robot to achieve stable forward flight, reaching a maximum velocity around 5.8 m s$^{-1}$ (Fig. 5a). In contrast, the wing’s aquatic mode is unable to generate sufficient lift during flight, causing the robot to lose altitude and crash. Furthermore, compared to the aquatic mode, the aerial mode enables the robot to carry an additional payload of 700 g during flight (total weight around 1550 g), demonstrating the enhanced lift performance achieved through asymmetric chordwise strokes and high spanwise stiffness.

In the aquatic swimming tests, to evaluate underwater propulsion performance, a dimensionless metric *EOF* (efficiency of forward motion) is defined as


(5) \begin{eqnarray*}
EOF = \frac{m g \bar{v}_x}{U \bar{I}},
\end{eqnarray*}

where $\bar{v}_x$ is the robot’s average forward velocity, $\bar{I}$ is the average current consumption, *m = 0.85* kg is the robot mass, *g = 9.8* m s$^{-2}$ is the gravity acceleration, and *U = 14.8* V is the battery voltage. As shown in Fig. 5b, at a low flapping frequency of 0.15 Hz, the aquatic mode achieves a 2% higher *EOF* while reducing current consumption by 13% compared with the aerial mode. It should be noted that, during free swimming, when wing flapping resistance exceeds body oscillation resistance, the motor output is mainly consumed by body oscillation rather than effective wing flapping, greatly reducing propulsion (see Movie S5). At a higher transmitter throttle of 40%, the aerial mode enters this ineffective flapping regime, whereas the aquatic mode still maintains effective flapping. Consequently, the aquatic mode improves *EOF* by 156% compared to the aerial mode, confirming its lower underwater flapping resistance and the effectiveness of the proposed mode-switching mechanism. As the throttle increases further, wing flapping in both modes becomes dominated by body oscillation, resulting in converging and degraded propulsion performance.

Since the flapping-wing robot relies on its tail wings for turning, and the effectiveness of tail wings is highly dependent on the propulsion of flapping wings, we further evaluate the turning performance of the robot in both aerial and aquatic environments (see Movie S6). The robot operates in aerial mode during flight tests and switches to aquatic mode during swimming tests. Similarly, the robot’s positions are recorded through motion capture system. As shown in Fig. 5c, during aerial flight at a flapping frequency of 3.00 Hz, the robot performs a turning maneuver of approximately *70*° in <1 s, with a turning radius around 0.8 m. While during aquatic swimming at a flapping frequency of 0.15 Hz (Fig. 5d), the robot achieves a *55*° turning angle in 15 s, with a turning radius around 1.5 m. These results demonstrate that the amphibious bimodal flapping wing generates sufficient propulsion for forward movement and turning maneuvers, enabling controllable operation of flapping-wing robots in both aerial and aquatic environments.

### Amphibious validations in the field

Amphibious locomotion of flapping-wing robots mainly involves two key capabilities: (i) efficient propulsion in both air and water; (ii) stable transition between water and air. However, direct water-to-air transition requires extremely high power density and launch velocity [40,41], exceeding the capabilities of current flapping-wing robots due to limitations in actuation power and structural strength [1]. This work focuses on improving amphibious propulsion and proposes an indirect water–ground–air transition strategy that exploits the robot’s payload capacity for practical amphibious operation.

**Figure 6.**
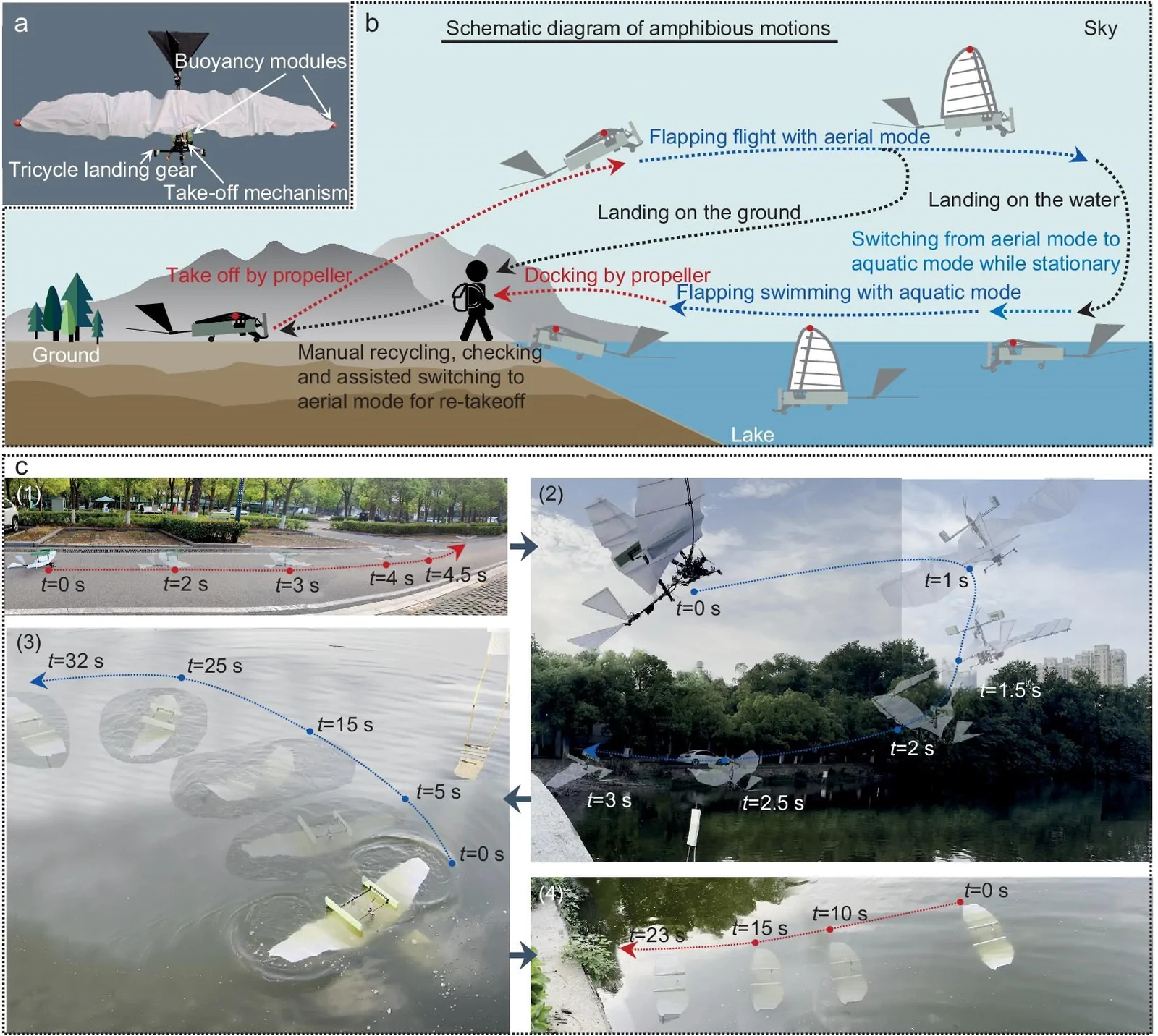
Field amphibious validations of the flapping-wing robot equipped with bimodal wings. (a) Additional auxiliary structures are incorporated to enhance the robot’s transition capabilities in aerial-aquatic environments, including a take-off mechanism, a tricycle landing gear, and buoyancy modules. (b) A conceptual motion strategy is proposed for amphibious operations. The robot initiates gliding take-off powered by the propeller of the take-off mechanism, then transitions to flapping-wing propulsion for efficient cruising in the air and in the water. The take-off mechanism also assists the robot in approaching the shore and landing on the ground, enabling manual recovery and relaunch. (c) Field tests of the actual robot validate the feasibility of key functionalities, such as propeller-assisted take-off, flapping-wing flight, flapping-wing swimming, and propeller-assisted shore approach.

Some auxiliary structures are integrated into the proposed flapping-wing robot to enable amphibious operation (see Fig. S16). As shown in Fig. 6a, a take-off mechanism is mounted at the front of the robot, where a brushless-motor-driven propeller provides burst thrust for launch. A tricycle landing gear is installed beneath the robot, allowing it to perform ground take-off similar to a fixed-wing aircraft. In addition, buoyancy modules provide a net buoyancy slightly greater than the robot’s weight during underwater operation. The total mass of these auxiliary structures is 220 g, remaining within the robot’s payload capacity. The robot’s waterproof design and control framework are provided in Figs S6 and S17, respectively.

The proposed indirect media-crossing procedure is illustrated in Fig. 6b. Initially, the robot takes off from the ground using the propeller. Subsequently, the take-off mechanism is deactivated, and the flapping mechanism is engaged to transition into more efficient flapping-based cruising. Upon completing aerial tasks, the robot can either return to the ground for recovery or land on the water surface to perform underwater operations. After landing on the water surface, the wings remain stationary while the mode-switching mechanism synchronously converts both wings to the aquatic mode, followed by efficient flapping-based swimming. After completing underwater tasks, the robot can rapidly approach the shore using the propeller and then be manually retrieved, inspected, and relaunched. Although this indirect media-crossing strategy cannot achieve fully autonomous water-to-air transition, it provides a feasible engineering solution under current technological limitations.

We validate the feasibility of the proposed amphibious motion strategy in outdoor scenarios (Fig. 6c and Movie S7). The designed robot is capable of taking off from an asphalt surface using the take-off mechanism and performing effective forward and turning maneuvers during flapping flight. After landing on the lake surface, the flapping wings also enable forward and turning swimming. Additionally, the propeller of the take-off module can propel the robot in the water, allowing it to approach the shore for recovery. However, switching the wings from the aquatic mode back to the aerial mode before relaunch may require manual assistance, as the weight of the wings may occasionally cause slight misalignment between the rigid spars and the spar bases. This can be readily corrected during manual recovery and inspection before the next take-off.

## CONCLUSIONS

In this study, inspired by birds and manta rays, we explore a bimodal flapping wing tailored for both aerial and aquatic environments. Drawing on the structural layouts and functional principles of bird wings and manta ray pectoral fins, we conducted their differences in skeletal stiffness and flapping strokes. By developing a lightweight mode-switching mechanism, spanwise and chordwise joints, the bimodal wing achieves repeatable stiffness and stroke switching. The dynamics test results indicate that the wing’s aerial mode achieves significant lift enhancement in the air, while its aquatic mode reduces consumption and improves thrust efficiency during underwater flapping. By comparing the propulsion differences between the two modes, we further confirm that the wing’s aerial mode achieves a larger payload during flight, while the aquatic mode reduces energy consumption and improves efficiency during swimming. Our work provides a feasible reference for the aerial-aquatic amphibious application of large-scale flapping-wing robots.

However, there are some limitations in our work. The proposed robot prioritizes aerial flight performance, sacrificing part of its underwater swimming capability (see Fig. S15). Future work can optimize the wing geometry and kinematics for more efficient underwater propulsion. In addition, the proposed amphibious operation relies on a ground-assisted transition between water and air, involving manual recovery and relaunch. Future research can further develop autonomous control and water-to-air transition strategies.

## Supplementary Material

nwag520_Supplemental_Files
