# Supplementary material for: Wings with stiffness and stroke variations enabling efficient flight and swimming for flapping-wing robots
Source: Natl Sci Rev. 2026 Aug 22;13(18):nwag520. doi: 10.1093/nsr/nwag520 (PMC13599785; doi:10.1093/nsr/nwag520)
Supplement: Supplementary file 1 [file nwag520_supplemental_files.zip › 04_Final_Supplementary_information.pdf]

Supplementary information

Wings with Stiffness and Stroke Variations Enabling Efficient Flight and Swimming for Flapping-Wing Robots.

Yang Xiang,† Zeyu Gong,† Chenyi Yang, Licheng Hou, Zhenwei Zhang, Yuhang Hong, Zhenfeng Gu, Le Gu, Ying Shi, Bo Tao,* Han Ding

State Key Laboratory of Intelligent Manufacturing Equipment and Technology, Huazhong University of Science and Technology, Wuhan, 430074, China.

SUPPLEMENTARY METHODS

Theoretical analysis of chordwise stroke variations

Since flapping motion involves complex three-dimensional fluid-structure interaction, it is challenging to achieve a perfect theoretical analysis of flapping dynamics. However, simplified models can be employed to investigate the relative influence trends of structural and kinematic parameters on dynamic performance and to identify approximate optimal parameter ranges. To simplify the analysis of how chordwise joint strokes affect flapping dynamics, two assumptions are made based on existing flapping dynamics calculation methods: i) the wing is assumed to be rigid, with only the deformation induced by the chordwise joint considered [1]. ii) the cross-section at 3/4 of the wing semi-span is selected as the equivalent plane to represent the overall semi-wing deformation [2].

The coordinate system and schematic used for the calculations are shown in fig. S5. The chord length of the wing surface that can rotate with the joint is defined as *c*, the flapping frequency as *f*, the semi-span length as *D*, time as *t*, the flapping angle as $\theta(t)$, and the passive chord rotation angle as $\phi(t)$. The calculation is nondimensionalized by scaling length with $c/2$, time with $1/f$, and velocity with $cf/2$. The *x*-axis is defined along the rotatable wing chord, where $x=-1$ corresponds to the rotation point of chordwise joint and $x=1$ corresponds to the trailing edge. The kinematic description of the equivalent cross-section during the flapping is given as follows:

$$h(x,t)=\frac{3D}{2c}\sin\theta+(x+1)\sin\phi. \tag{1}$$

For the sinusoidal function sin *F*(*t*), its first-order Taylor expansion at *F*(*t*) = 0 can be approximated as *F*(*t*). Considering that both θ and ϕ are periodic oscillatory functions with maximum amplitudes within 45°, the maximum deviation resulting from the first-order Taylor expansion of $\sin\theta$ and $\sin\phi$ is approximately 4.52°, which is acceptable for the calculation. Therefore, the equation (1) can be simplified as:

$$h(x,t)=\frac{3D}{2c}\theta+(x+1)\phi. \tag{2}$$

We simulated the chordwise joint motion during flapping by setting two parameters: i) The initial angle of chordwise joint ϕ_0, determines the offset of the joint motion stroke. ii) The torsional spring stiffness of chordwise joint κ_c, affects the dynamic response of the joint. According to the reference [1], the passive chordwise stroke follows the torque balance principle:

$$J_c\ddot{\phi}=-\kappa_c(\phi-\phi_0)+N_{fc}+N_{fi}. \tag{3}$$

Where, $J_c=\frac{1}{3}\rho_s bc^3$ denotes the rotational inertia of the chord about the joint, ρ_s is the wing surface density, and *b* is the wing thickness. N_{fc} represents the moment exerted on

the chordwise section by the fluid, and N_{ic} denotes the moment due to flapping acceleration on the section. Defining the fluid density as ρ and the oncoming flow velocity as V_∞. The equation (3) can be nondimensionalized both sides by $\rho c^4/16$:

$$\frac{16}{3}R_c\ddot{\phi} = -4K_cV^2f^2(\phi-\phi_0)+\tilde{N}_{fc}+\tilde{N}_{ic}. \tag{4}$$

Here, $R_c = \dfrac{b\rho_s}{c\rho}$ denotes the ratio of wing to fluid inertia, $K_c = \dfrac{\kappa_c}{\rho {V_\infty}^2c^2}$ is the spring forces as compared to fluid forces, $V = \dfrac{2V_\infty}{cf}$ is the dimensionless oncoming flow velocity, $\tilde{N}_{fc}$ denotes the dimensionless fluid-induced torque, and $\tilde{N}_{ic}$ denotes the dimensionless flapping acceleration torque. $\tilde{N}_{ic}$ can be directly derived from the flapping motion [1]:

$$\tilde{N}_{ic} = \frac{6R_cD}{c}\ddot{\theta}. \tag{5}$$

However, the fluid-induced torque $\tilde{N}_{fc}$ is a coupled quantity influenced by fluid forces, torsional deformation, and the flapping motion, it therefore requires analysis of the surrounding flow field. We define a fluid domain as a unit circle centered at $x=0$, while an acceleration potential $\vartheta = \varphi + i\psi$ exists in this domain [3]. φ is Prandtl's acceleration potential, as grad φ gets the acceleration field of the flow. ψ is defined by the Cauchy–Riemann equations, which can be solved as:

$$-\frac{\partial\psi}{\partial x} = \left(\frac{\partial}{\partial t}+V\frac{\partial}{\partial x}\right)^2 h(x,t). \tag{6}$$

According to the results in [1], the expressions for φ and ψ are related as follows:

$$\begin{cases} \psi = \dfrac{1}{2}a_0 + a_1x + a_2x^2 \\ \varphi = \dfrac{1}{2}a_0\sqrt{\dfrac{1-x}{1+x}} + a_1\sqrt{1-x^2} + a_2x\sqrt{1-x^2} \end{cases}. \tag{7}$$

Here, a_0, a_1, and a_2 are undetermined coefficients. Substituting equation (2) into (6) and expanding the result into a power-series form analogous to equation (7) yields expressions for φ and ψ. The pressure difference between the upper and lower wing surfaces can be simplified as $p(x,t) = 2\varphi$, so that the fluid-induced torque $\tilde{N}_{fc}$ is expressed as:

$$\tilde{N}_{fc} = \int_{-1}^{1} p(x,t)\times(x+1)dx. \tag{8}$$

By substituting the results of equations (5) and (8) into the equation (4), with the boundary conditions $\phi(0)=\phi_0$ and $\dot{\phi}(0)=0$, the function of passive rotation angle $\phi(t)$ can be obtained. Subsequently, the dimensionless thrust $\tilde{T}$, lift $\tilde{L}$, and power $\tilde{P}$ generated by flapping can be calculated according to [3] as follows:

$$\tilde{T}=\tilde{T}_p+\tilde{T}_s, \tilde{T}_p=\int_{-1}^{1} p\frac{\partial h}{\partial x}dx, \tilde{T}_s=\frac{a_0^2}{64\pi f^2V^2}. \tag{9}$$

$$\tilde{L}=\int_{-1}^{1} pdx, \tilde{P}=\int_{-1}^{1} p\frac{\partial h}{\partial t}dx. \tag{10}$$

Since $\tilde{L}$, $\tilde{T}$, and $\tilde{P}$ are time-varying periodic functions, their equivalent values can be obtained by integrating over a single period t_p:

$$\bar{\tilde{L}}=\frac{\int_0^{t_p}\tilde{L}dt}{t_p}, \bar{\tilde{T}}=\frac{\int_0^{t_p}\tilde{T}dt}{t_p}, \bar{\tilde{P}}=\frac{\int_0^{t_p}\tilde{P}dt}{t_p}. \tag{11}$$

These equivalent values can be further scaled by the flapping frequency *f* and amplitude *A* to obtain the lift coefficient C_L, thrust coefficient C_T, and power coefficient C_P:

$$C_L=\frac{f\bar{\tilde{L}}}{4\pi^3A^2}, C_T=\frac{f\bar{\tilde{T}}}{4\pi^3A^2}, C_P=\frac{f\bar{\tilde{P}}}{4\pi^3A^2}. \tag{12}$$

The corresponding calculation results are shown in fig. S5. The theoretical analysis indicates that, in both aerial and aquatic environments, the downward-biased asymmetric stroke ($\phi_0<0$) generates greater lift, while the symmetric stroke ($\phi_0=0$) achieves higher thrust performance. Excessively high or low torsional spring stiffness κ_c reduces overall dynamic performance. These results are basically consistent with experimental measurements. It should be noted that, although this simplified calculation model cannot fully capture effects such as vortices and wake interactions in real flapping, it is feasible for analyzing the relative influence of joint parameters on flapping dynamics.

Theoretical analysis of spanwise bending stiffness variations

Similarly, the influence of the spanwise joint bending stiffness on dynamic performance can be analyzed using the same simplified calculation framework introduced above. Two assumptions are applied in the calculation: i) the wing is assumed to be rigid, with only the deformation induced by the spanwise joint considered. ii) the cross-section at flapping spar is selected as the equivalent plane to represent the overall semi-wing deformation.

The calculation coordinate system and schematic are shown in fig. S6. The driving spar of the wing is divided into three segments: an inner segment of length D_1, a variable-stiffness spanwise joint of length D_2, and an outer segment of length D_3, so that the total semi-span is $D=D_1+D_2+D_3$. We defined the flapping frequency as *f*, time as *t*, and the flapping angle

as $\theta(t)$. The calculation is nondimensionalized by scaling length with $D_3/2$, time with $1/f$, and velocity with $D_3 f/2$. The x_o-axis is defined along the outer segment span, with $x_o=-1$ at the junction between the outer segment and the spanwise joint, and $x_o=1$ at the tip of outer segment. During flapping, the bending of the spanwise joint is represented by a cantilever-beam model. The beam coordinate system $\{x_b,\delta\}$ is defined with x_b along the inner segment span and δ normal to the inner-span direction. The external loading on the spanwise joint may be approximated by an equivalent bending moment N_e applied at the end of the joint by the outer wing. Under this approximation, the deflection curve $\delta(x_b,t)$ and the bending angle $\tau(x_b,t)$ of the joint are given by the standard cantilever-beam expressions for an end moment:

$$\delta(x_b,t)=\frac{N_e(t)x_b^{\ 2}}{2\kappa_s},\ \tau(x_b,t)\approx\frac{\partial\delta}{\partial x_b}=\frac{N_e(t)x_b}{\kappa_s}. \tag{13}$$

Here, $\kappa_s=E_sI_s$ denotes the bending stiffness of the spanwise joint, where E_s is the elastic modulus and I_s is the area moment of inertia. Assuming that the spanwise joint undergoes small deflection, the horizontal coordinate at the joint tip can be approximated as $x_b=D_2$. By nondimensionalizing the tip deflection δ_e with respect to $D_3/2$, the nondimensional tip deflection $\tilde{\delta}_e(t)$ and bending angle $\tau_e(t)$ in beam model can be expressed as:

$$\tilde{\delta}_e=\frac{N_eD_2^2}{\kappa_sD_3},\ \tau_e=\frac{N_eD_2}{\kappa_s}. \tag{14}$$

Based on the principle of coordinate transformation, the kinematic equation of the outer wing segment during the flapping process can be expressed in the spatial coordinate as follows:

$$h(x_o,t)=(x_o+1)\sin(\tau_e+\theta)+\frac{2(D_1+D_2)}{D_3}\sin\theta+\tilde{\delta}_e\cos\theta. \tag{15}$$

We observe that the term $\tilde{\delta}_e\cos\theta$ in equation (16) is strongly coupled. Performing a first-order Taylor expansion analogous to equation (2) for simplification still leads to difficulties in solving the resulting expression. Using geometric relationships, equation (15) can be further approximated as:

$$h(x_o,t)=(x_o+1)\sin(\tau_e+\theta)+\frac{2D_1}{D_3}\sin\theta+\frac{2D_2}{D_3}\sin\left(\frac{\tau_e}{2}+\theta\right). \tag{16}$$

Perform a first-order Taylor expansion at 0 point for each sine term in equation (16):

$$h(x_o,t)=(x_o+1)(\tau_e+\theta)+\frac{D_2}{D_3}\tau_e+\frac{2D_1+2D_2}{D_3}\theta. \tag{17}$$

During flapping, the spanwise joint should also satisfy the moment balance among the elastic torque generated by bending, the inertial torque from flapping, and the fluid-induced torque. Following the equality in equation (3), the balance equation of spanwise joint can be formulated as:

$$J_s \ddot{\tau}_e = -N_e + N_{fs} + N_{is}. \tag{18}$$

Where, $J_s = \frac{\rho_s b D_3^3}{3}$ represents the rotational inertia of the outer wing segment swinging with the spanwise joint, N_{fs} represents the moment exerted on the spanwise section by the fluid, and N_{is} denotes the moment due to flapping acceleration on the section. Combining with the expressions in equation (14), $N_e = \frac{\kappa_s D_3}{D_2^2}\tilde{\delta}_e$ and $\ddot{\tau}_e = \frac{D_3}{D_2}\ddot{\tilde{\delta}}_e$. Similarly, both sides of the equation are divided by $\rho D_3^4 / 16$ for nondimensionalization:

$$\frac{16 D_3 R_s}{3 D_2}\ddot{\tilde{\delta}}_e = -4 K_s V^2 f^2 \frac{D_3}{D_2^2}\tilde{\delta}_e + \tilde{N}_{fs} + \tilde{N}_{is}. \tag{19}$$

$R_s = \frac{b\rho_s}{D_3 \rho}$ denotes the ratio of wing to fluid inertia, $K_s = \frac{\kappa_s}{\rho V_\infty^2 D_3^2}$ is the elastic forces as compared to fluid forces, $V = \frac{2V_\infty}{D_3 f}$ is the dimensionless oncoming flow velocity, $\tilde{N}_{fs}$ denotes the dimensionless fluid-induced torque, and $\tilde{N}_{is}$ denotes the dimensionless flapping acceleration torque. A unit circle is constructed at $x_o = 0$ as the computational domain for the flow field, within which the acceleration potential $\vartheta = \varphi + i\psi$ can also be applied. Here, φ and ψ can be calculated by substituting equation (17) into equations (6) and (7). The pressure difference between the upper and lower surfaces of the wing is $p = 2\varphi$. Accordingly, the fluid-induced moments $\tilde{N}_{fs}$ and $\tilde{N}_{is}$ can be expressed as:

$$\tilde{N}_{fs} = \int_{-1}^{1} p(x_o, t) \times (x_o + 1) dx_o, \ \tilde{N}_{is} = \frac{8 R_s D_1}{D_3}\ddot{\theta}. \tag{20}$$

By substituting Eq. (20) into Eq. (19) with the boundary conditions $\tilde{\delta}_e(0) = 0$ and $\dot{\tilde{\delta}}_e(0) = 0$, the tip deflection of the spanwise joint in the beam coordinate can be solved. It should be noted that due to simplifications and approximations in multiple calculation steps, the obtained deflection must be multiplied by a correction factor around $\alpha = \frac{f D_3}{3 V_\infty}$ to match the experimentally observed deformation. Based on the above calculations, the complete form of equation (15) can be obtained, and the dynamics performance for evaluation can be computed similarly using equations (9), (10), (11), and (12).

The corresponding calculation results are summarized in fig. S6. The results indicate that, in both aerial and aquatic flapping, higher spanwise stiffness generates greater lift and thrust

but also requires higher power consumption. In aerial flapping, increasing stiffness beyond a certain value will no longer significantly improves dynamic performance. However, higher stiffness also implies greater structural weight, so an optimal spanwise stiffness can be selected from a lightweight design perspective. In aquatic flapping, there exists a peak in thrust efficiency, which can serve as a basis for evaluating the optimal spanwise stiffness for aquatic operation. The theoretical predictions of the spanwise bending stiffness effect and the optimal parameter range are basically consistent with experimental results.

Measurements and processing of dynamics

During dynamic tests, the lift L and thrust T are measured by a 6-axis force/torque sensor (KWR75B, Kunwei Sensing Technology Co.,Ltd), and the current consumption C is measured by a current monitor. For each set of experiments, multiple flapping cycles are conducted at each flapping frequency. To minimize random disturbances, the collected data are first processed using a low-pass filter with a cutoff frequency of 20 Hz, followed by periodic fitting using a 6th-order Fourier Series:

$$FS = a_0 + \sum_{i=1}^{6}\left(a_i \cos(i\omega t) + b_i \sin(i\omega t)\right). \tag{21}$$

where the coefficients a_0, a_i, and b_i are the unknown variables to be estimated during fitting, while ω corresponds to the flapping frequency f used in the experiments and is expressed as $\omega = 2\pi f$.

This periodic fitting approach helps to obtain the statistical values of multiple measurement cycles and convert them into a single representative function. Furthermore, evaluation metrics, such as equivalent lift $\overline{L}$, thrust $\overline{T}$, and current $\overline{C}$, can be computed by integrating the fitted periodic function over one cycle, enabling consistent performance comparisons across different parameter configurations within a unified analytical framework. The equivalent power $\overline{P}$ is obtained by multiplying the equivalent current $\overline{C}$ by the operating voltage U.

$$\overline{L} = f\int_0^{1/f} L dt, \overline{T} = f\int_0^{1/f} T dt, \overline{C} = f\int_0^{1/f} C dt. \tag{22}$$

$$\overline{P} = U\overline{C}. \tag{23}$$

Statistical analysis of dynamics tests

The fitted values are used to represent dynamic forces and power consumption in Figs. 4 and 5. To assess the reliability of the results, we analyzed the discrepancies between the periodic fitting results over multiple flapping cycles and the corresponding measurements from individual cycles. Error bars are plotted in Fig. S13 shown that the data deviation tends to increase with flapping frequency. In general, both the maximum and minimum error trends remain consistent with the fitted values, and do not affect the results of optimal stroke and stiffness in these tests.

Lift and thrust efficiency calculation in dynamics tests

Since our dynamic tests are conducted under zero-flow conditions, the commonly used Froude efficiency, which requires a predefined free stream velocity for calculation, cannot be directly applicable as a performance metric in our evaluation. Based on a modified Froude efficiency that used to assess the thrust performance of bio-inspired robotic fish [4], we conduct an analysis of the corresponding expression:

$$\eta = \frac{TV}{TV + P} = 1 - \frac{1}{1 + V\frac{T}{P}}. \tag{24}$$

where T denotes the thrust generated during motion, P represents the power consumption, and V is the forward velocity of robot. From the analysis of formula expression, it can be observed that under a given flow velocity V, a higher T/P ratio corresponds to a higher Froude efficiency η. Therefore, in our zero-flow tests, we adopt T/P as the thrust efficiency metric, and generalize this approach to lift by using L/P as the lift efficiency metric.

Experimental set-up in motion tests

The aerial flight tests are performed in a 6 m (width) × 8 m (length) × 5 m (height) space, while four marks are attached to the robot's body. A motion capture system (Opti-track, NaturalPoint Inc.) is used to record positions and velocities of the marks, which achieves a native frame rate of 180 FPS and a 3-D accuracy of ±0.1 mm. For the aquatic swimming tests, the robot is operated in a 3 m (width) × 4 m (length) × 1.5 m (depth) pool, and four marks are attached to the robot's body similarly. A motion capture system (Mars4H UW, NOKOV Science & Technology Co., Ltd.) with a native frame rate of 180 FPS and a 3-D accuracy of ±0.15 mm is used to record positions and velocities of the marks. Moreover, the current consumption during motion is recorded by the INA226 chip.

Waterproofing of the robot

The robot's structures are made of anodized metal gears, carbon plates, carbon spars, carbon rods, and 3D-prints, which can be directly used underwater. For electrics and battery, the conventional approach typically involves enclosing them in an acrylic chamber sealed with O-rings to ensure waterproofing. However, this approach introduces signific additional mass and geometry to the robot, reducing the aerial flight capability. Therefore, we adopt a more lightweight and compact waterproofing method using silicone encapsulation.

Firstly, heat-shrinkable sleeves appropriately sized to the electronic components are placed around them respectively. Then, silicone is injected into the gaps between the component and sleeve. Finally, the assembly is heated with a heat gun, causing the sleeve to shrink tightly around the component. During this process, the silicone forms a reliable seal around the electronics, and any excess silicone is automatically squeezed out, minimizing unnecessary weight (see Fig. S6).

References

[1] M. Moore, Analytical results on the role of flexibility in flapping propulsion, *J. Fluid Mechan.* 2014, **757**, 599-612.

[2]C. Ruiz, J. Acosta, A. Ollero, Optimal elastic wing for flapping-wing robots through passive morphing. *IEEE Robot. Autom. Lett.* 2022, **8**, 608-615.
[3]T. Wu, Swimming of a waving plate. *J. Fluid Mech.* 1961, **10**, 321-344.
[4]C. Hemelrijk, D. Reid, H. Hildenbrandt, J. Padding, The increased efficiency of fish swimming in a school, *Fish. Fish.* 2015, **16**, 511-521.

SUPPLEMENTARY FIGURES

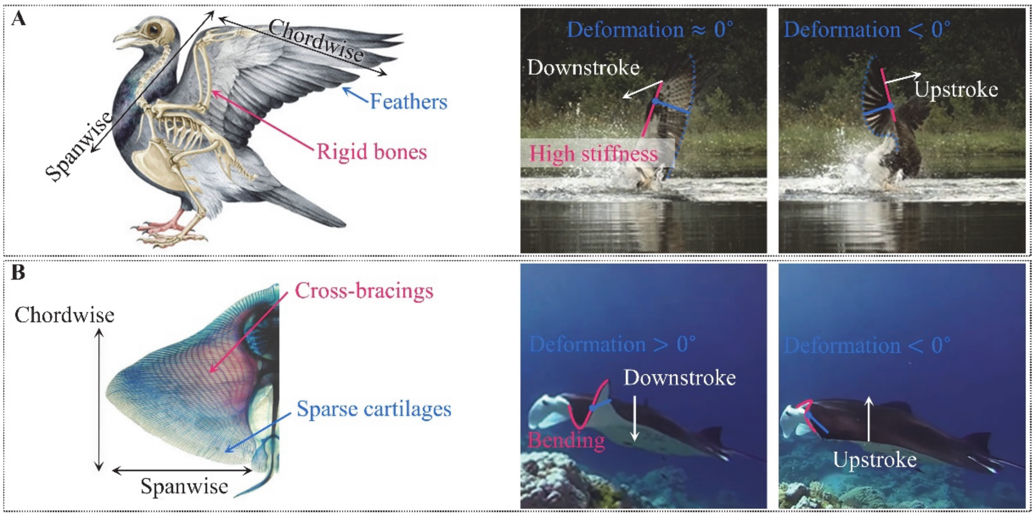

Fig. S1. Comparison between bird wings and manta ray fins. (**A**) Bird wings are composed of rigid bones and feathers. The bones are distributed along spanwise and actively drive the flapping motion of the wings, while the feathers are oriented along chordwise. The high spanwise stiffness ensures that the wings maintain sufficient structural strength to effectively disturb the airflow during flapping. Meanwhile, the feathers undergo asymmetric deformation during the flapping cycle (deformation angle is approximately zero during downstroke, whereas the deformation angle becomes negative during upstroke). This asymmetry enables the wing to generate positive lift with a larger effective area during downstroke and reduce negative lift with a smaller area during upstroke. (**B**) The pectoral fins of manta rays consist of multiple cross-bracings and cartilages, with the structural density decreasing toward the fin edges. During flapping, the fins exhibit notable spanwise flexibility. This flexibility helps concentrate motion at the fin tips, thereby reducing flapping resistance. In chordwise, the fins undergo approximately symmetric deformation throughout the flapping cycle (deformation angle is positive during downstroke while it tends to negative during upstroke). Such symmetry is effective in canceling lift and generating thrust.

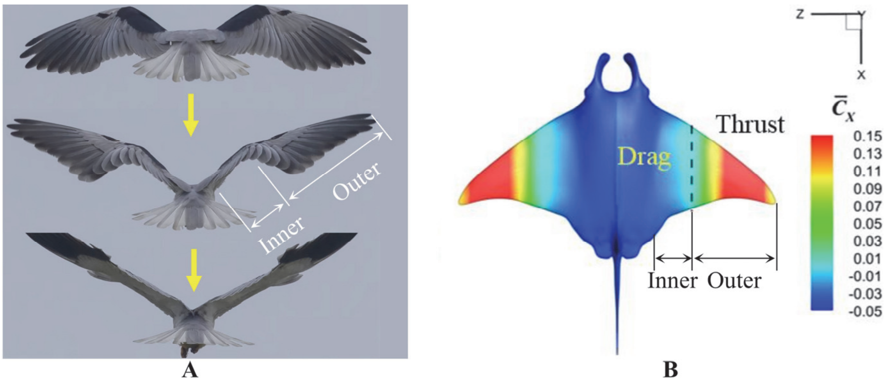

Fig. S2. Deformation regions of biological flapping wings and fins. (**A**) During bird flapping, significant folding deformation mainly occurs in the outer 70%-80% span region of the wing. (**B**) During manta ray swimming, compliant deformation is also concentrated in the outer 70% span region of the pectoral fin.

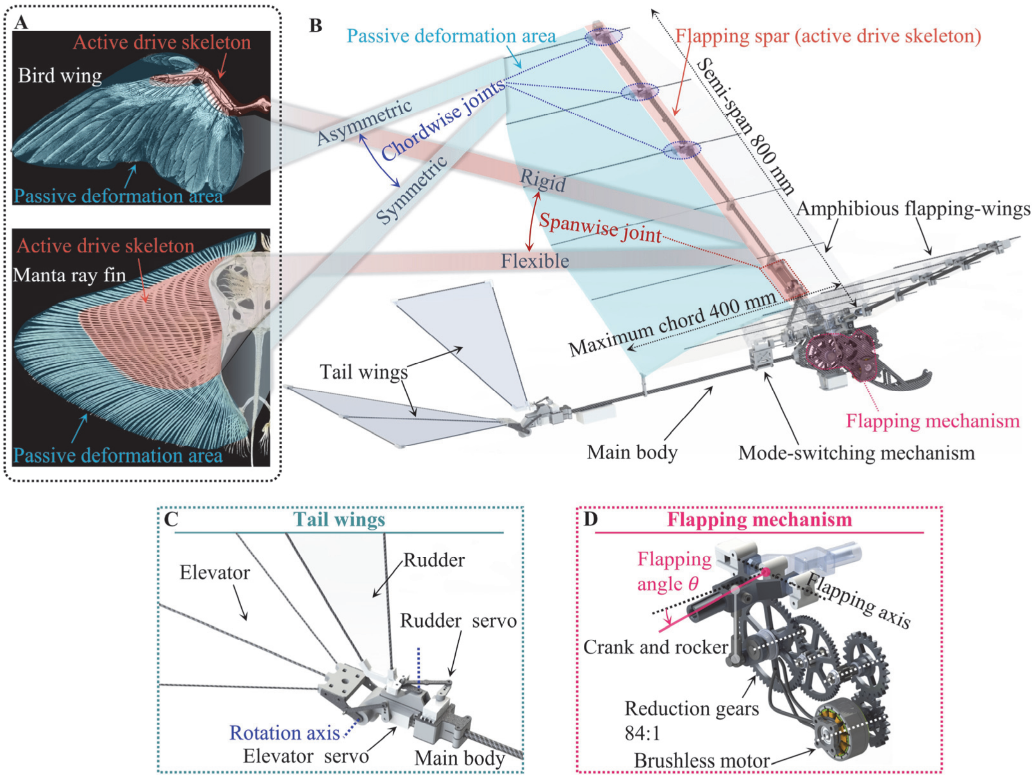

Fig. S3. Structural details of the bimodal flapping-wing robot equipped with the bimodal flapping wings. (**A**) According to the biomimetic inspiration, the bird wings and manta ray fins can be simplified as systems driven by a primary skeletal region, while surrounding tissues attached to the skeleton undergoing passive deformation. (**B**) The bimodal flapping-wing robot mainly consists of four parts, main body, mode-switching mechanism, a pair of bimodal flapping wings, and tail wings. (**C**) The tail wings consist of an elevator and a rudder, where the elevator controls the pitch attitude of the robot and the rudder is responsible for heading control. (**D**) The flapping mechanism is driven by a brushless motor (VELOX V2808, T-motor) with a weight around 60 g. A four-stage gear reduction system is used to amplify the motor's output torque by a factor of 84. The reduced gears are connected to the flapping wings via a crank-rocker mechanism, which transmits the mechanical power to drive the wing's flapping.

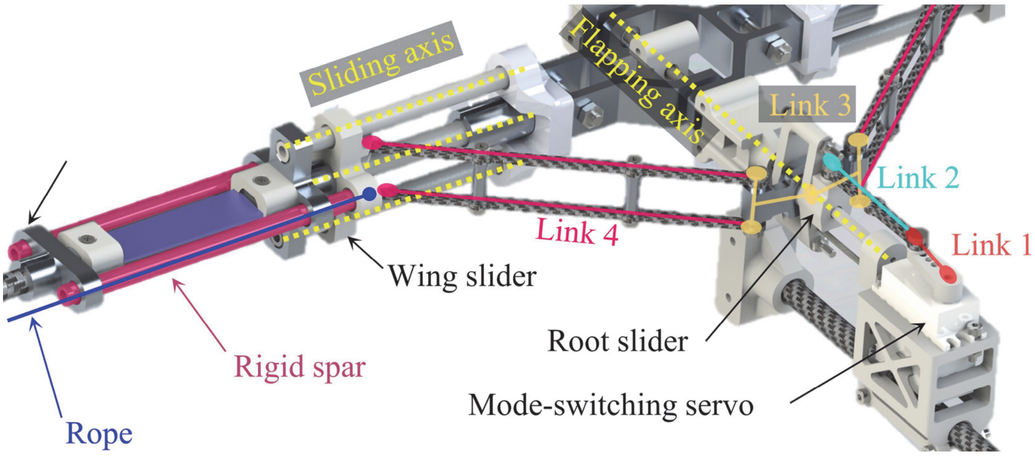

Fig. S4. Structural details of the mode-switching mechanism. The mode-switching mechanism, mounted on the robot's main body, consists of a servo, four-stage links, a root slider and two wing sliders (one for each wing). Links 1 and 2 connect the servo to the root slider, converting the servo's rotational output into the linear displacement of the root slider along an axis collinear with the wing's flapping axis. Link 3 is symmetrically hinged on both left and right sides of the root slider, allowing coordinated sliding motion without interfering with wing flapping. Link 4 transmits the root slider's displacement to each wing slider, driving them toward either the wingtip or the wing root. This design enables a single servo to synchronously control both wings' mode switching.

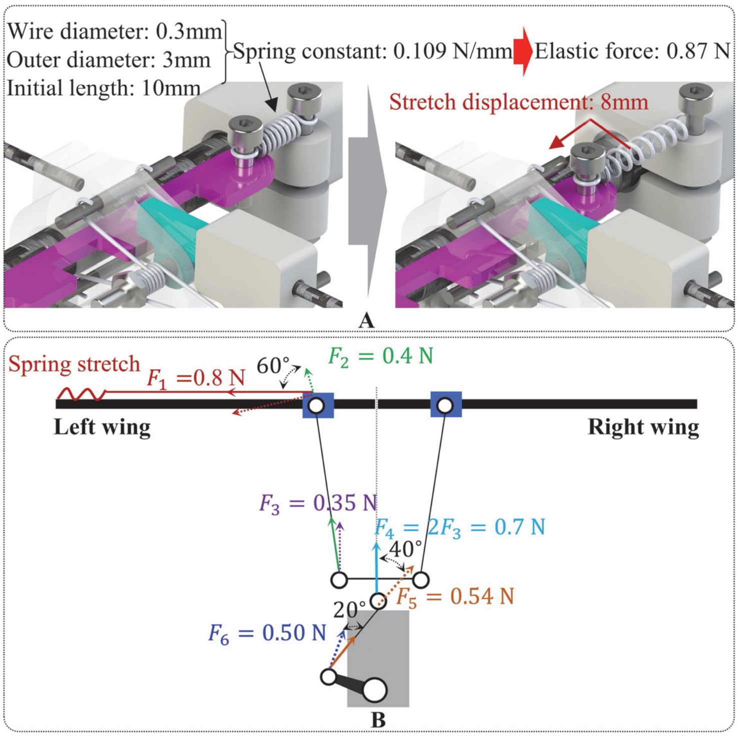

Fig. S5. Force analysis of the mode-switching mechanism. (**A**) Specifications of the wingtip spring and its extension in aquatic mode. (**B**) Force transmission from the wingtip to the servo through the linkage. The current of mode-switching servo during aquatic mode holding is below 10 mA, indicating very low power consumption with negligible impact on endurance.

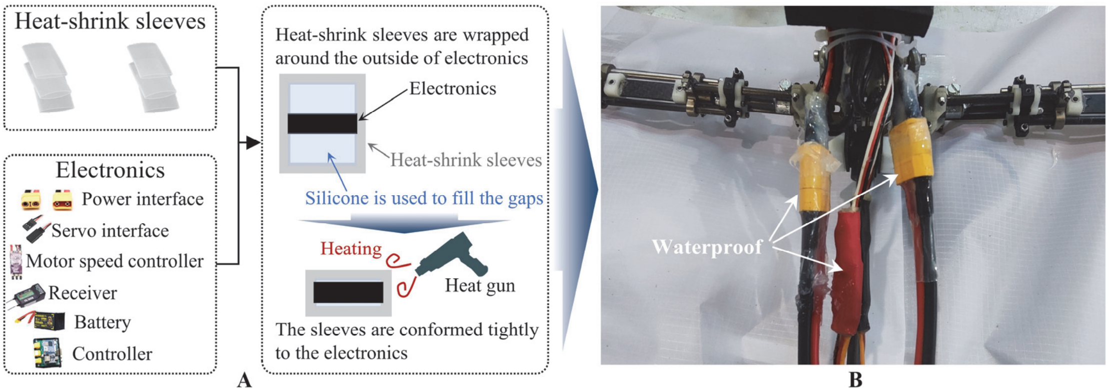

Fig. S6. Schematic of the robot waterproofing. (**A**) Unlike conventional waterproof method through acrylic compartments, we adopted a more compact and lightweight waterproof processing. Heat-shrink sleeves are placed around the robot's electronics, while the gaps and edges between the sleeve and electronics are filled with waterproof silicone. Subsequently, the assembly is heated using a heat gun, causing the sleeves to shrink tightly around the electronics. During this process, excess silicone is adaptively expelled, thereby reducing unnecessary weight. (**B**) Post-treatment physical view of the electronics. Under corresponding underwater tests, the waterproofing method effectively prevented water ingress and electrical short-circuits.

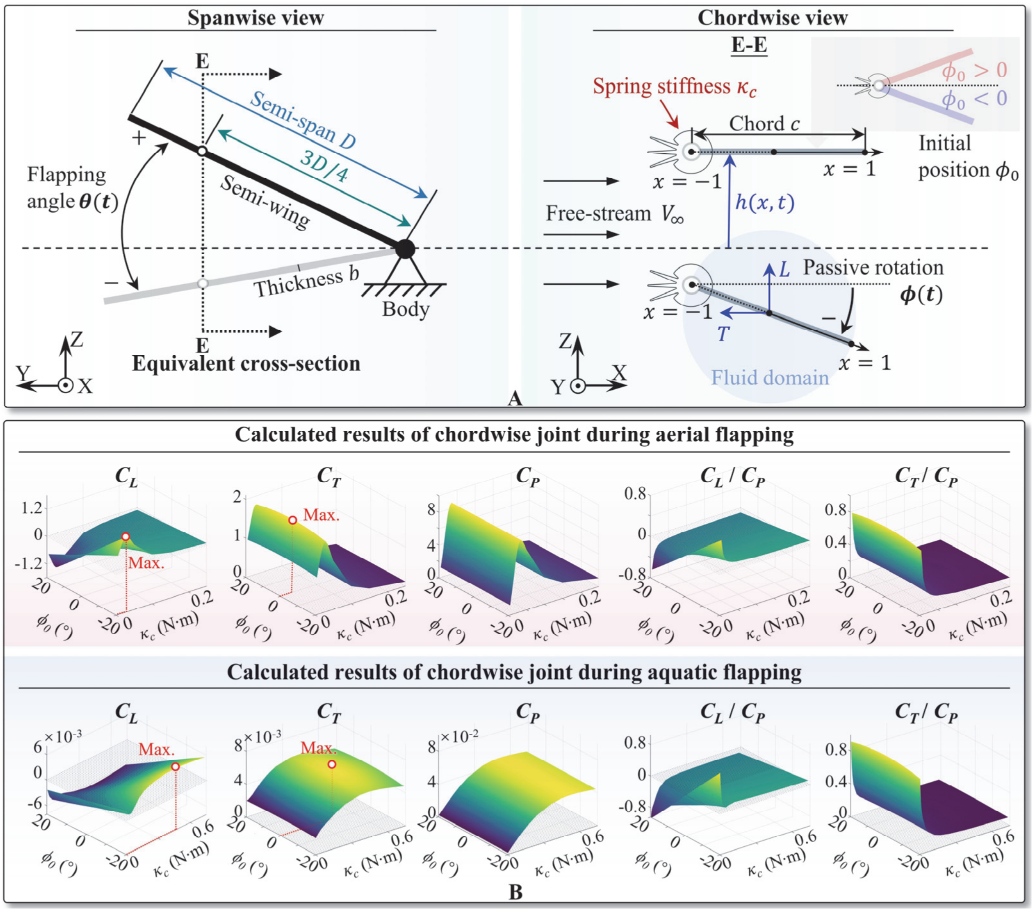

Fig. S7. Computational principles and results for evaluating the dynamics of the chordwise joint with stroke variations. (**A**) The coordinate system and variables used in the analysis. The wing is assumed as rigid, and only the rotational deformation of the chordwise joint is considered. The cross-section at 3D/4 is selected as the equivalent plane for simplified computation. Under a prescribed flapping motion with angle $\theta(t)$, the joint undergoes a passive rotation $\phi(t)$, determined jointly by the initial angle ϕ_0 (which sets the stroke offset) and the torsional spring stiffness κ_c (which determines the dynamic response). (**B**) Based on the robot's operating conditions, results are presented for $f = 3$ Hz and $V_\infty = 6$ m/s in flight and $f = 0.15$ Hz and $V_\infty = 0.15$ m/s in swimming. For both aerial and aquatic flapping, asymmetric strokes with $\phi_0 < 0$ (larger projected area during downstroke than upstroke) significantly enhance lift, whereas symmetric strokes with $\phi_0 = 0$ improve thrust. Thus, switching the chordwise-joint stroke enables adjustments of flapping-wing dynamics for amphibious adaptation. The optimal torsional spring stiffness κ_c is about 0.03 N·m for flight and 0.44 N·m for swimming. In practice, prioritizing lightweight structure and stable aerial performance, the aerial optimal stiffness can be chosen for the chordwise-joint design. It should be noted that this simplified computational model cannot fully capture real flapping phenomena, but it provides a feasible method to analyze the relative influence trends and optimal range of joint parameters. The obtained results are generally consistent with experimental measurements.

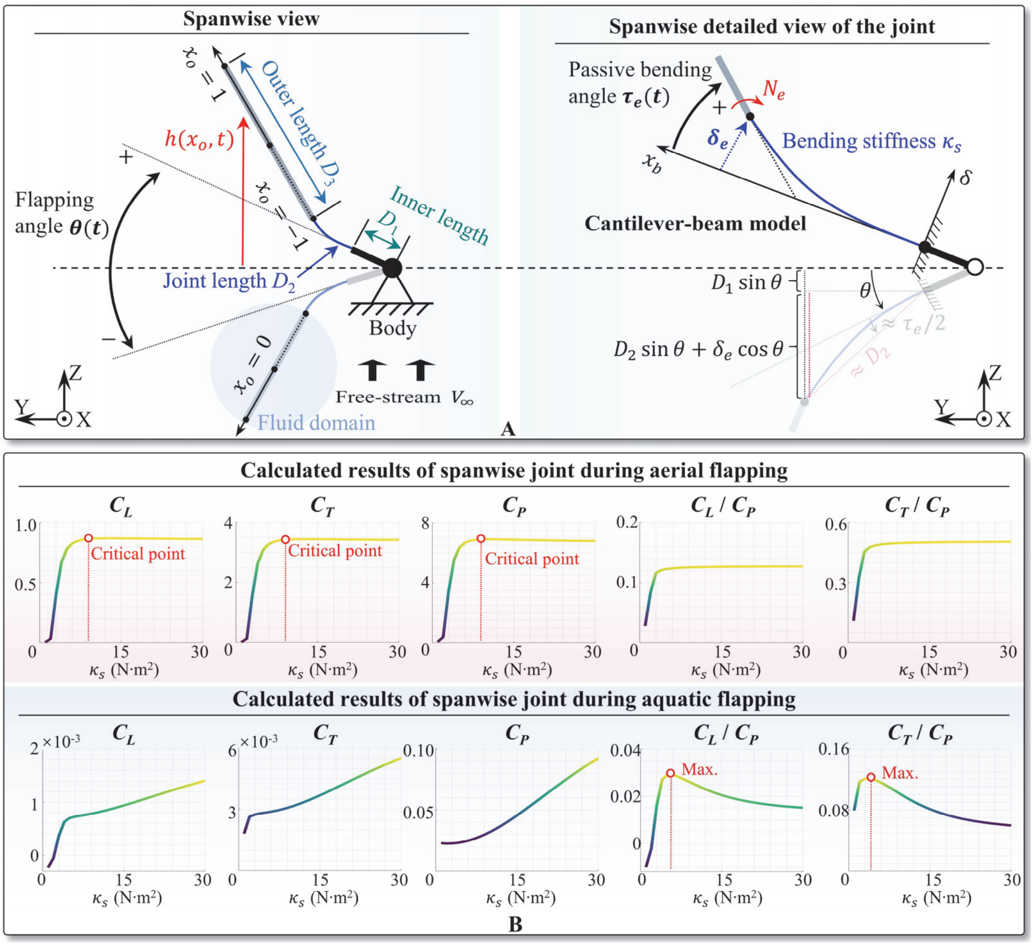

Fig. S8. Computational principles and results for evaluating the dynamics of the spanwise joint with bending stiffness variations. (**A**) The coordinate system and variables used in the analysis. Similarly, the wing is assumed as rigid, and only the bending deformation of the spanwise joint is considered. The cross-section at flapping spar is selected as the equivalent plane for simplified computation. (**B**) Accordingly, results are presented for $f = 3$ Hz and $V_\infty = 6$ m/s in flight and $f = 0.15$ Hz and $V_\infty = 0.15$ m/s in swimming. The computational results for both aerial and aquatic flapping show that forces and power consumption increase with the bending stiffness of the spanwise joint. In aerial flapping, once the bending stiffness exceeds a critical value of about $\kappa_s = 9$ N·m^2, further increases produce negligible changes in performance because the wing's spanwise deformation becomes minimal. Since higher stiffness typically adds weight, this critical value provides a practical reference for lightweight design. In aquatic flapping, however, power consumption grows faster with stiffness than lift or thrust. Consequently, both C_L / C_P and C_T / C_P reach a maximum value at $\kappa_s = 5$ N·m^2, indicating that moderate spanwise flexibility is beneficial for low-power, high-efficiency swimming. While the simplified model cannot capture all real flapping effects, it provides a feasible method to analyze the influence trends and optimal parameter ranges of joint, which generally align with experimental measurements.

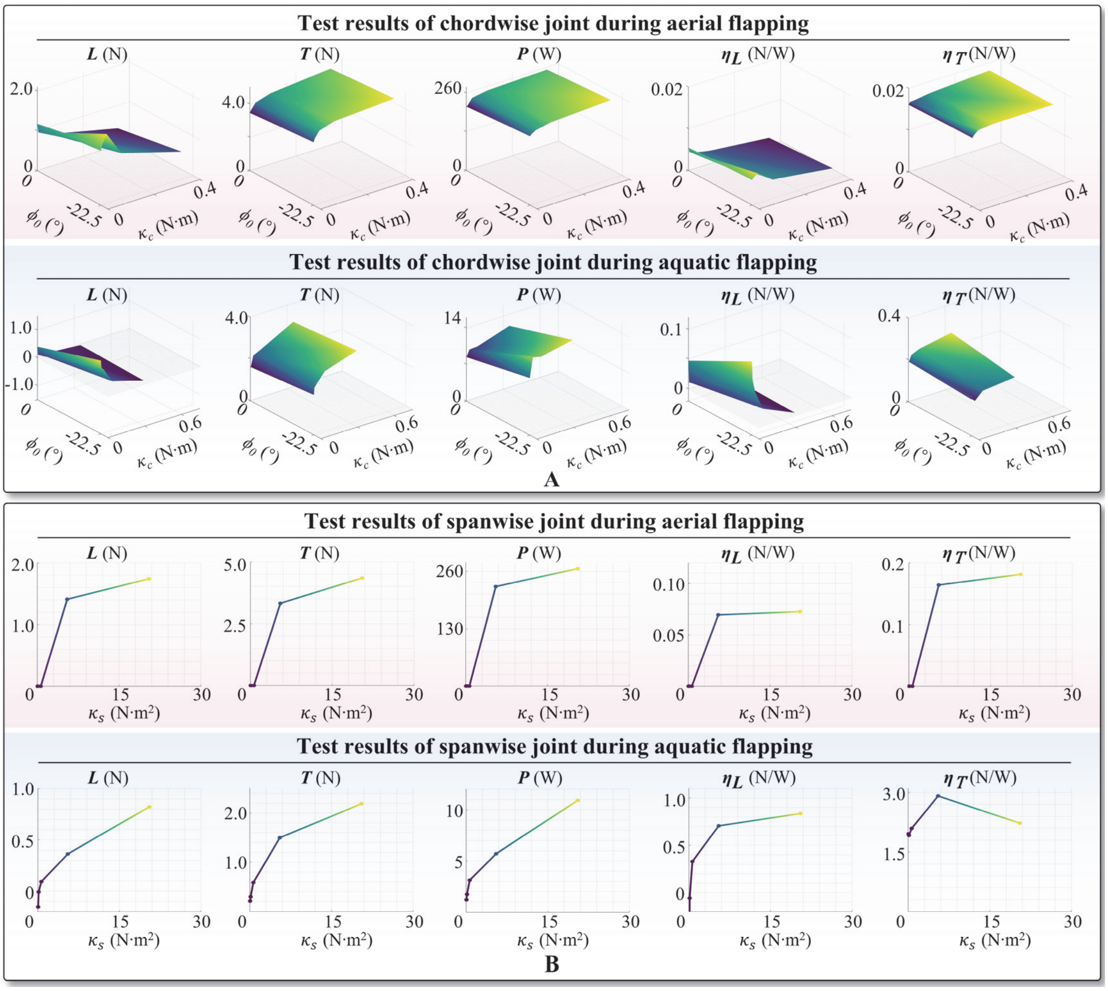

Fig. S9. Experimental results compared with theoretical analysis. (**A**) Experimental data of chordwise joints with different stroke and spring stiffness under aerial flapping at $f = 3$ Hz and aquatic flapping at $f = 0.15$ Hz. From the optimization objectives of maximizing lift for aerial flight and minimizing power consumption/improving thrust efficiency for underwater swimming, the experimental results indicate that asymmetric stroke with $\phi_0 < 0°$ and $\kappa_c = 0.025$ N·m provides the optimal aerial lift performance (theoretical results: asymmetric stroke with $\phi_0 < 0°$ and $\kappa_c = 0.025$ N·m), while the symmetric stroke with $\phi_0 = 0°$ and $\kappa_c = 0.350$ N·m achieves the optimal underwater thrust efficiency (theoretical results: symmetric stroke with $\phi_0 = 0°$ and $\kappa_c = 0.440$ N·m). (**B**) Experimental data of spanwise joints with different bending stiffness under aerial flapping at $f = 3$ Hz and aquatic flapping at $f = 0.15$ Hz. The experimental results indicate that aerodynamic performance would no longer significantly increase when approximately $\kappa_s > 12$ N·m^2 (theoretical results: $\kappa_s > 9$ N·m^2), while the optimal underwater thrust efficiency occurs at $\kappa_s = 5.50$ N·m^2 (theoretical results: $\kappa_s = 5$ N·m^2). Overall, the theoretical and experimental results are consistent in both effect trends and optimal parameter ranges.

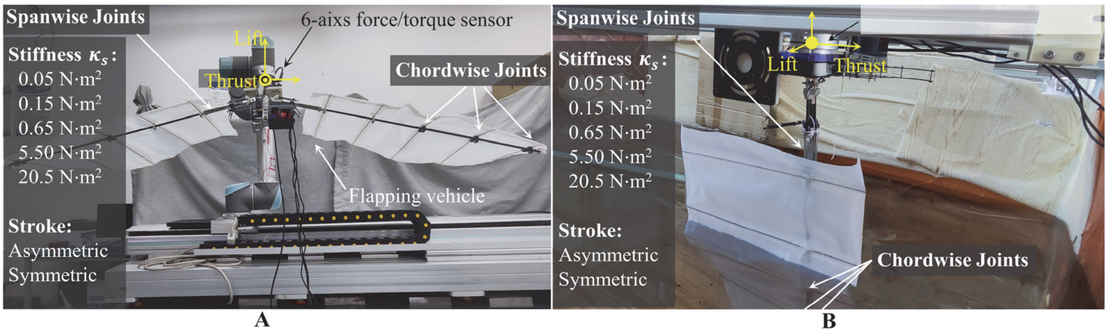

Fig. S10. Dynamic test scenario. (**A**) Aerial flapping tests. The flapping vehicle is mounted on a robotic arm via a 6-axis force/torque sensor (KWR75B, Kunwei Sensing Technology Co., Ltd), with a pair of bimodal flapping wings attached on either side of the vehicle. Tests are conducted across various parameter settings for the spanwise and chordwise joints. For each parameter setting, multiple cycles are tested at frequencies ranging from 0.5 Hz to 3.0 Hz. (**B**) Aquatic flapping tests. A bimodal flapping wing is fixed to the pool frame through the 6-axis force/torque sensor. Similarly, different configurations of spanwise and chordwise joint parameters are evaluated. For each parameter setting, multiple cycles are tested at frequencies ranging from 0.05 Hz to 0.30 Hz.

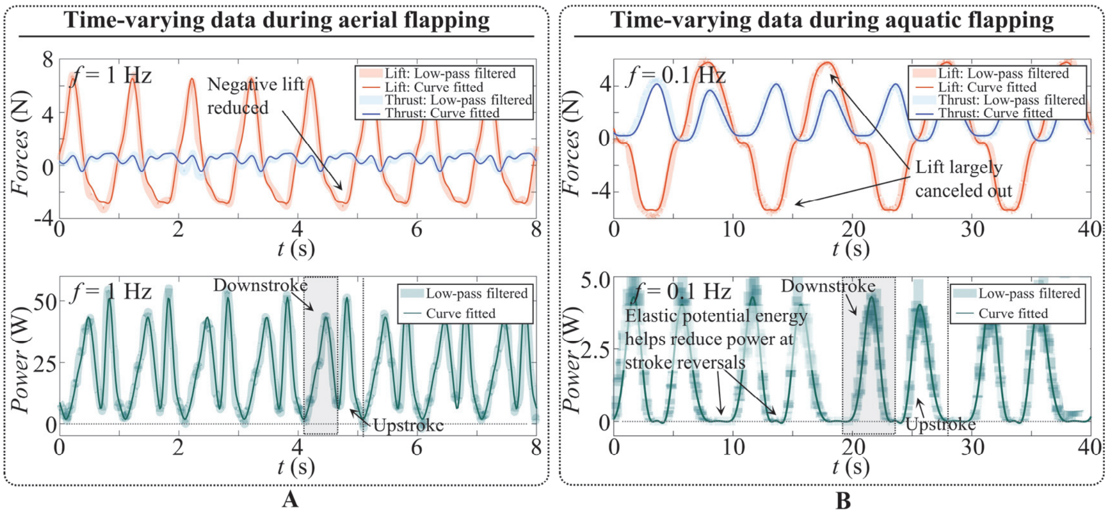

Fig. S11. Samples of time-varying data in dynamic tests. (**A**) Lift, thrust, and power data in aerial flapping tests at $f = 1$ Hz, with 20.5 N·m^2 spanwise stiffness and asymmetric chordwise stroke. The negative lift during upstroke is significantly reduced, effectively enhancing the lift performance over a flapping cycle. In addition, due to the larger wing area in downstroke compared to the upstroke, the total energy consumption during downstroke is greater. (**B**) Lift, thrust, and power data in aquatic flapping tests at $f = 0.1$ Hz, with 5.50 N·m^2 spanwise stiffness and symmetric chordwise stroke. The lift is largely cancelled out during downstroke and upstroke, while thrust is relatively increased compared to lift. Since the wing areas during upstroke and downstroke are approximately equal, the total energy consumption during both phases is essentially comparable. Moreover, appropriate spanwise flexibility enables beneficial deformation during flapping, as well as allows stored elastic energy to assist shape recovery during stroke reversals, thereby reducing overall flapping energy consumption.

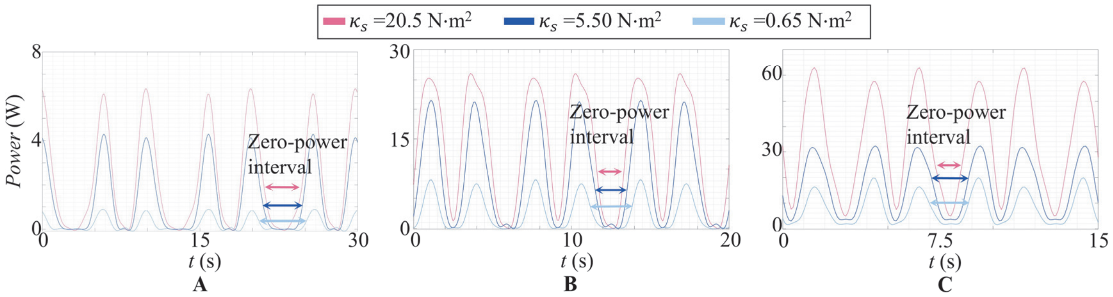

Fig. S12. Time-varying data of power consumption under different spanwise bending stiffness during underwater tests. (**A**) Power profiles at $f = 0.1$ Hz. (**B**) Power profiles at $f = 0.15$ Hz. (**C**) Power profiles at $f = 0.2$ Hz. Consistent trends are observed across all tested frequencies: with increasing spanwise stiffness κ_s , the peak power during flapping increases, while the zero-power interval during stroke reversal becomes shorter.

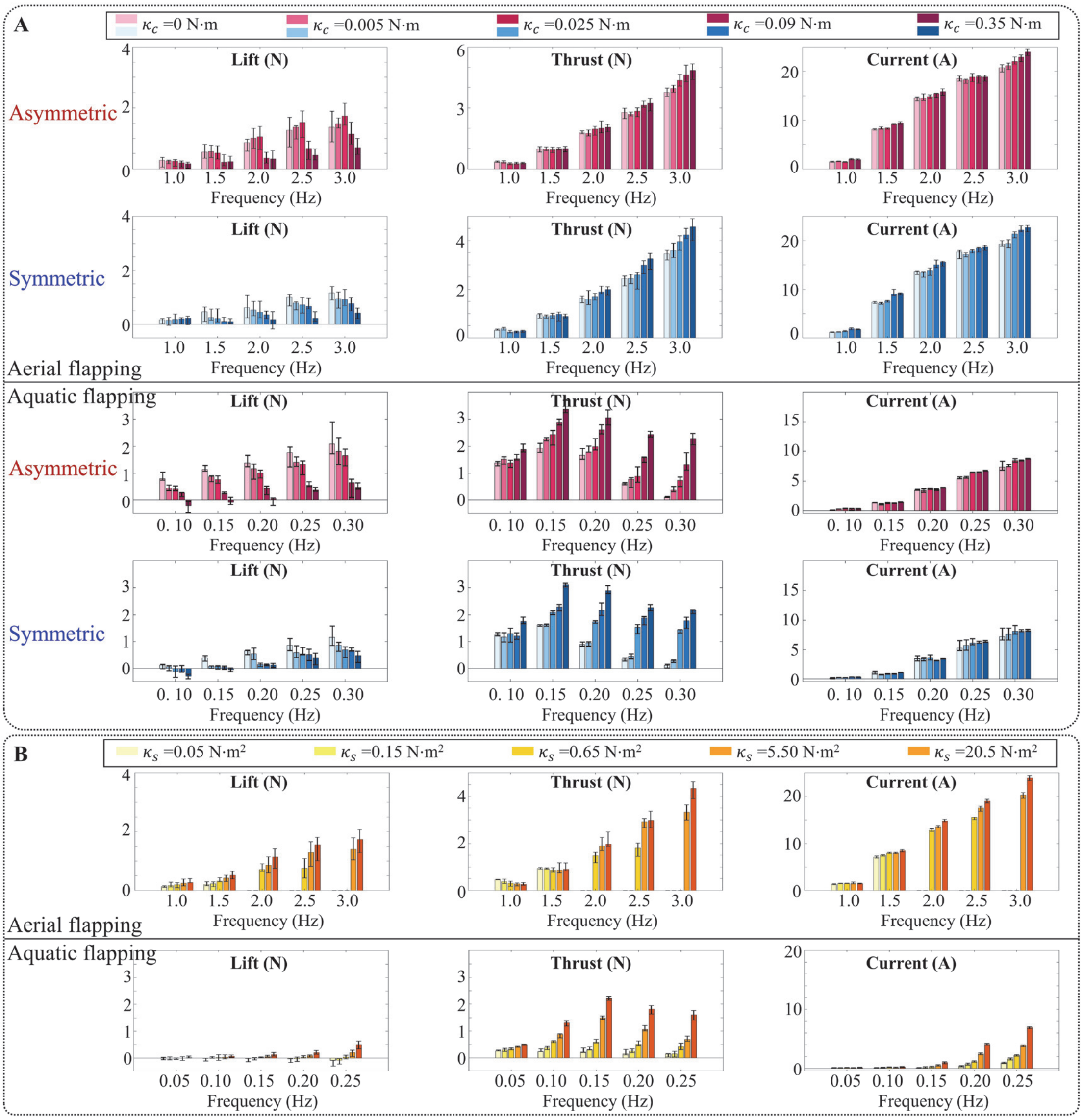

Fig. S13. Statistical measures in dynamic tests. (**A**) Error statistics between the fitted values and single-cycle measurements of the chordwise joint tests. Since lift is more sensitive to variations in wing area, it is more affected by random disturbances during testing. This is reflected in the fact that lift exhibits larger errors compared to thrust and power consumption. As the flapping frequency increases, the errors caused by random disturbances also tend to rise. Overall, both the maximum and minimum errors follow the same trend as the fitted values, and do not affect the optimal results of the chordwise stroke or torsional spring stiffness. (**B**) Error statistics between the fitted values and single-cycle measurements of the spanwise joint tests. Similarly, errors induced by random disturbances are more pronounced in lift and increase with flapping frequency. Nevertheless, both the maximum and minimum errors exhibit trends consistent with the fitted values, and thus do not affect the conclusions regarding the influence of spanwise stiffness on dynamics.

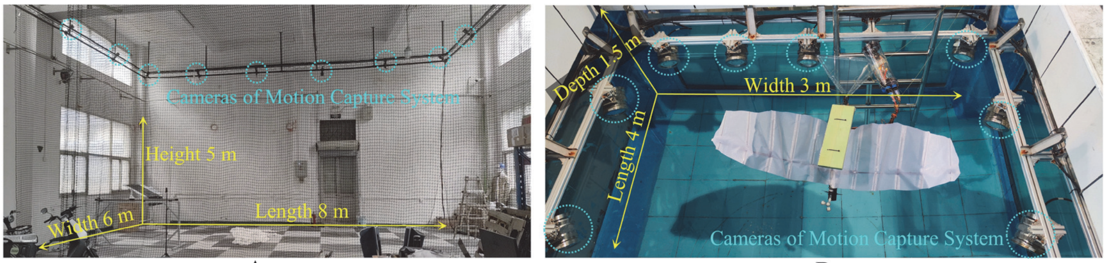

A B

Fig. S14. Experimental setups for motion tests. (**A**) The test site for aerial motion is measured 8 m in length, 6 m in width, and 5 m in height. A motion capture system consisting of 16 cameras is used to track the 3D velocity and position of markers attached to the robot. (**B**) Aquatic swimming tests are conducted in a pool measuring 4 m in length, 3 m in width, and 1.5 m in depth. The motion capture system with 8 cameras is deployed to capture the 3D motion data of the robot. The motor current consumption is recorded using an onboard INA226 chip.

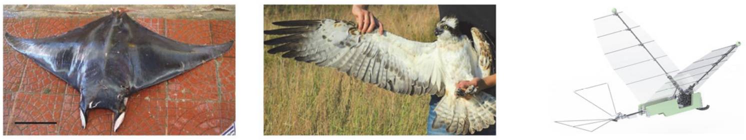

**Metrics**	**Manta ray (*Mobula japanica*)**	**Bird (*Osprey*)**	**The proposed robot**
**Wing geometry**	Span: 2180 mm<br>Chord: 975 mm	Span: 1600 mm<br>Chord: 280 mm	Span: 1600 mm<br>Chord: 400 mm
**Aspect ratio**	2.2	5.7	4
**Body mass**	65 kg	1.4 kg	0.85 kg
**Wing loading**	—	0.32 g/cm^2	0.13 g/cm^2
**Flapping frequency**	0.2~0.6 Hz	2~4 Hz	Aerial: 3~4 Hz<br>Aquatic: 0.1~0.3 Hz
**Speed**	0.05~1.5 m/s	8~15 m/s	Aerial: 6 m/s<br>Aquatic: 0.1 m/s

Fig. S15. Comparison of the proposed amphibious flapping-wing robot with biological birds and manta rays. In the practical design of the proposed robot, aerial flight imposes more stringent constraints, such as lightweight structure and stable high-frequency flapping. Therefore, the overall wing geometry primarily follows that of bird wings to ensure effective and stable flight performance, while the flapping principles of manta ray fins are integrated to enhance aquatic propulsion. As a result, the underwater flapping frequency and swimming speed of the robot still exhibit a gap compared to those of real manta rays. However, our biomimetic concept does not aim to directly replicate the geometric morphology of birds or manta rays. Instead, it focuses on mimicking their compliant fluid-structure interaction mechanisms in air and water, thereby providing a feasible design approach for efficient amphibious propulsion in flapping-wing robots.

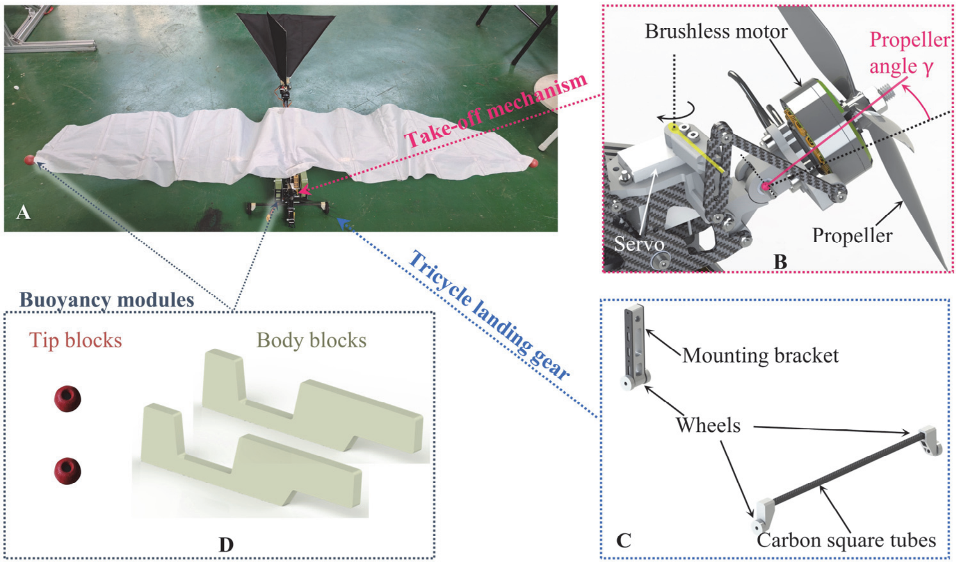

Fig. S16. Auxiliary structures used for outdoor amphibious validation. (**A**) Assembled auxiliary structures. (**B**) The take-off mechanism is installed at the head of the robot and consists of a brushless motor, a propeller, a servo, and links. The brushless motor drives the propeller to generate burst thrust, while the servo adjusts the propeller's pitch angle through the links, enhancing the flexibility of the propeller. (**C**) The tricycle landing gear is mounted on the bottom of the robot, enabling taxiing by maintaining contact with the ground via wheels. (**D**) The buoyancy module consists of body and tip blocks, providing a total buoyancy slightly greater than the robot's weight while ensuring that the wings remain fully submerged during underwater operation.

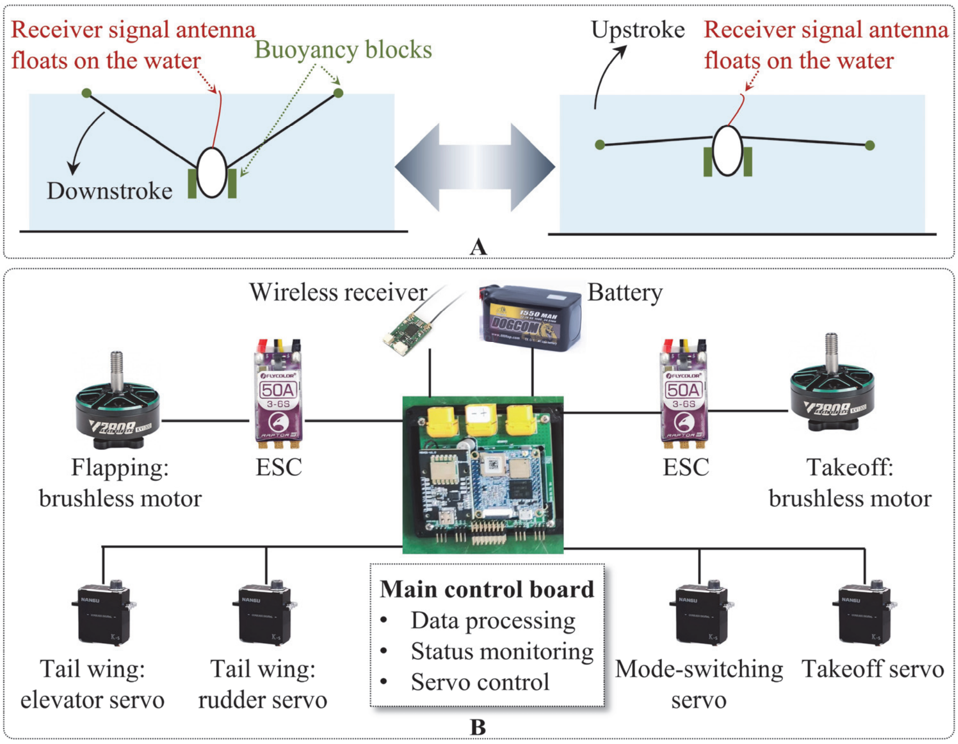

Fig. S17. Wireless control framework of the amphibious flapping-wing robot. (**A**) Receiver signal antenna floats on the water surface during swimming to enable signal transmission stability. This approach may be limited in deep-water scenarios. (**B**) Control system of the proposed robot. The control board decodes the input signals and drives two ESCs to control the flapping and takeoff brushless motors, respectively. The control signals are also transmitted to four waterproof servos for real-time actuation of the tail fin, mode-switching mechanism, and other components.

SUPPLEMENTARY MOVIES

Movie S1. Biological inspirations of the bimodal flapping wing. This video shows the flapping principles of bird wings and manta ray pectoral fins, with the bio-inspired bimodal flapping wing design based on stiffness and stroke variations. The wing's aerial mode mimics bones rigidity and feather asymmetric deformation of birds, while the aquatic mode mimics the skeleton flexibility and fin symmetrical deformation of manta rays. By switching between these two modes, flapping wings can adapt effectively to both air and water environments, enhancing propulsion performance for efficient flight and swimming in flapping-wing robots.

Movie S2. Demonstration of different wing mode. This video shows the effects of different spanwise stiffness and chordwise stroke on wing deformation during flapping. In the aerial mode, the spanwise joints exhibit large bending stiffness with negligible deformation, while the chordwise joint generates an asymmetric rotational stroke. In the aquatic mode, the spanwise joint exhibits certain flexibility to produce appropriate deformation adapting to the fluid, and the chordwise joint generates a symmetric rotational stroke. Further reducing the flexibility of spanwise joints will increase its bending deformation.

Movie S3. Physical demonstration of mode switching. This movie shows the mode-switching details in mode-switching mechanism, spanwise joints, and chordwise joints.

Movie S4. Dynamic tests in aerial and aquatic environments. This movie shows examples of aerial and aquatic dynamic tests. In aerial flapping, the high skeleton stiffness paired with asymmetric stroke enables the wing to generate dynamics, while the excessive skeleton flexibility leads to structural failure. In aquatic flapping, the appropriate skeleton flexibility with symmetric stroke helps reduce resistance and enhance efficiency, while excessive skeleton flexibility will prevent effective force transmission and result in inefficient flapping.

Movie S5. Motion tests under different modes. This movie illustrates the aquatic mode of the bimodal flapping wings cannot maintain effective flight, occurring crash during tests. While the aerial mode achieves stable flight with a maximum payload of 700 g. During underwater swimming, the aquatic mode exhibits a reduced velocity but also consumes less energy, thereby enhancing overall efficiency. Meanwhile, this movie also confirms that an excessive spanwise flexibility will lead to a significant decrease in robot propulsion, while aerial mode is more likely to cause the body oscillation rather than effective wing flapping.

Movie S6. Turning tests of the bimodal flapping wing robot. This video shows the turning trajectory of the robot equipped with bimodal flapping wings, demonstrating that the propulsion generated by the wings is sufficient to achieve controllable operations.

Movie S7. Outdoor validations. This movie shows the motion performance of the bimodal flapping-wing robot in outdoor environments. By carrying some auxiliary structures, the robot achieves ground take-off, flapping flight, flapping swimming, and propeller-assisted shoreline approach, indirectly achieving medium-crossing between aquatic and aerial environments for potential applications.
